# Computational Analysis of CD46 Protein Interaction with SARS-CoV-2 Structural Proteins: Elucidating a Putative Viral Entry Mechanism into Human Cells

**DOI:** 10.3390/v15122297

**Published:** 2023-11-23

**Authors:** Pavel Vassiliev, Evgenii Gusev, Maria Komelkova, Andrey Kochetkov, Maria Dobrynina, Alexey Sarapultsev

**Affiliations:** 1 Laboratory for Information Technology in Pharmacology and Computer Modeling of Drugs, Research Center for Innovative Medicines, Volgograd State Medical University, 39 Novorossiyskaya Street, Volgograd 400087, Russia; akocha@mail.ru; 2 Institute of Immunology and Physiology, Ural Branch of the Russian Academy of Science, 106 Pervomaiskaya Street, Yekaterinburg 620049, Russia; gusev36@mail.ru (E.G.); mzurochka@mail.ru (M.D.); 3 Russian-Chinese Education and Research Center of System Pathology, South Ural State University, 76 Lenin Prospekt, Chelyabinsk 454080, Russia; mkomelkova@mail.ru

**Keywords:** alternative receptors, CD46, cellular invasion, computational techniques, SARS-CoV-2 entry, structural proteins, therapeutic treatments

## Abstract

This study examines an unexplored aspect of SARS-CoV-2 entry into host cells, which is widely understood to occur via the viral spike (S) protein’s interaction with human ACE2-associated proteins. While vaccines and inhibitors targeting this mechanism are in use, they may not offer complete protection against reinfection. Hence, we investigate putative receptors and their cofactors. Specifically, we propose CD46, a human membrane cofactor protein, as a potential putative receptor and explore its role in cellular invasion, acting possibly as a cofactor with other viral structural proteins. Employing computational techniques, we created full-size 3D models of human CD46 and four key SARS-CoV-2 structural proteins—EP, MP, NP, and SP. We further developed 3D models of CD46 complexes interacting with these proteins. The primary aim is to pinpoint the likely interaction domains between CD46 and these structural proteins to facilitate the identification of molecules that can block these interactions, thus offering a foundation for novel pharmacological treatments for SARS-CoV-2 infection.

## 1. Introduction

COVID-19, caused by SARS-CoV-2, remains a pressing global health concern despite the existence of effective vaccines [1]. The primary entry mechanism of SARS-CoV-2 involves the interaction of the viral receptor-binding domain (RBD) of the spike protein (SP) with the human angiotensin-converting enzyme 2 (ACE2) receptor on the cell surface. The molecular intricacies of this ACE2-mediated viral entry have been comprehensively reviewed [2,3]. Intriguingly, SARS-CoV-2 has the capability to infect cells or organs that do not express ACE2, indicating the potential role of alternative receptors; this subject is discussed in [4,5]. ACE2-independent entry presents a promising avenue for evading antibodies targeting the spike RBD [6]. The exploration of receptors and their associated targets stands as a substantial step forward in the quest to find solutions for mitigating SARS-CoV-2 infection [7]. These findings highlight the potential existence of receptors, distinct from ACE2, capable of enabling alternative entry mechanisms for SARS-CoV-2 into human cells. Notably, various receptors with established roles in pathogen entry, as well as the modulation of cellular functions and immune responses, are currently under examination [4].

One receptor of interest is CD46. Due to its ubiquitous expression, complement regulatory activities, immune-modulating signaling functions, and internalization mechanisms, CD46 emerges as a likely candidate for interaction with various pathogens [8]. It has been described as a “pathogens’ magnet” for its role as a receptor for numerous pathogens, including different adenovirus species B and D, measles virus, herpesvirus 6A, cytomegalovirus, and several bacteria [9]. Interaction of CD46 with pathogens on T-cells contributes to an immunosuppressive phenotype in T-cells [10]. Specifically, CD46-mediated signaling leads to the co-expression of immunosuppressive IL-10 in Th1 cells, facilitating a self-regulating and contracting phase of the immune response [11].

Consequently, it is highly plausible that the interaction between SARS-CoV-2 and the human membrane cofactor protein CD46 could represent a yet-to-be-explored mechanism contributing to severe COVID-19 complications. This opens a potential pathway for the development of new therapeutic strategies targeting CD46 to manage severe SARS-CoV-2 infections.

Interestingly, SARS-CoV-2 structural proteins in COVID-19, including the S, M, and N proteins, can accumulate in the blood in a complex with hemoglobin and its metabolites [12,13]. The S protein can hyperactivate immune cells via Toll-like receptors [14]. These facts substantiate the need to examine the binding ability of SARS-CoV-2 structural proteins with other immune-associated receptors, including CD46.

The hypothesis of this study is that the ubiquitously expressed receptor CD46 may interact with E, M, N, and S proteins, thereby potentially participating in the virus’s entry into target cells. Moreover, CD46′s interaction with other SARS-CoV-2 structural proteins could potentially contribute to the pathogenesis of COVID-19 by exerting an immunosuppressive effect.

The objective of this study is to construct heterodimeric complexes between the human membrane cofactor protein CD46 and the structural proteins EP, MP, NP, and SP of SARS-CoV-2, with the aim to identify the most probable regions for their interaction.

This study comprises several key stages:

(1) Compilation of a dataset of experimental 3D models of the specified proteins and identification of their valid subunits.
(2) Homology modeling of full-length 3D structures for these proteins and selection of their best 3D models.
(3) Construction of multiple 3D models for CD46 protein complexed with EP, MP, NP, and SP proteins, and identification of the most relevant models.
(4) An analysis of binding regions in the adequate 3D models of protein–protein complexes and identification of key binding amino acids.
(5) Selection of the best 3D models for the heterodimeric complexes of human CD46 protein with EP, MP, NP, and SP proteins of SARS-CoV-2, and identification of the most probable interaction regions.

As a result of the conducted research, valid full-length 3D models of heterodimeric complexes between the human CD46 protein and the EP, MP, NP, and SP proteins of SARS-CoV-2 were constructed for the first time. The spectrum of key binding amino acids in these complexes was determined, and the most probable regions of interaction between the human CD46 protein and the EP, MP, NP, and SP proteins of SARS-CoV-2 were identified.

It is assumed that the obtained 3D models, information on key binding amino acids, and data on interaction regions in heterodimeric complexes of CD46 with EP, MP, NP, and SP proteins of SARS-CoV-2 can serve as a basis for the search for chemical compounds that block these interactions. It is likely that such substances may form the basis for the development of novel therapeutic agents to treat severe SARS-CoV-2 infections.

## 2. Materials and Methods

In this study, the primary amino acid sequences of human CD46 and EP, MP, NP, and SP proteins of SARS-CoV-2, as well as experimental 3D models of these five proteins, were used as materials.

The calculations were performed using high-performance computing equipment with a total capacity of approximately ~44 Tflops.

The main findings are presented in the main text of the article, and more detailed information, if needed, is provided in the Supplementary Information in the form of tables, which are numbered with the prefix “S”.

The main strain of SARS-CoV-2 considered in this study is the wild-type Wuhan strain M908947 (Wuhan-Hu-1) [15]. This strain serves as a “standard” and has been the basis for the development of most currently used vaccines [16].

The SARS-CoV-2 genome encodes 31 proteins [17], which can be grouped as follows:

(1) Structural proteins: envelope EP, membrane MP, nucleocapsid NP, spike SP. These proteins are predominantly located on the external surface of SARS-CoV-2 and deter-mine the virus’s shape.
(2) Non-structural proteins: NsP1, NsP2, NsP3, NsP4, NsP5, NsP6, NsP7, NsP8, NsP9, NsP10, NsP11, NsP12, NsP13, NsP14, NsP15, NsP16. After entering human cells, these proteins perform various functions, although the functions of some of these proteins are not yet fully understood.
(3) Proteins encoded by open reading frames: ORF1a, ORF1b, ORF3a, ORF3b, ORF6, ORF7a, ORF7b, ORF8, ORF9b, ORF10, ORF14. Like the non-structural proteins, these proteins also perform various functions after entering human cells, and the functions of some of these proteins are not yet defined.

It is believed that during the entry of the virus into human cells, the protein SP predominantly interacts with its receptors, while the interactions of proteins EP, MP, and NP with these receptors are limited due to steric hindrance associated with the S protein [18]. However, in the case of SARS-CoV-2 entry into human cells via fusion, all structural proteins of the virus can be integrated into the cytoplasmic membrane of the infected cell and may potentially function as cellular receptors or co-receptors. Therefore, for this study, the structural proteins EP, MP, NP, and SP were chosen as the most likely candidates for interaction with human CD46.

Human CD46 protein forms 16 different isoforms due to alternative splicing. For the purposes of this study, the most commonly occurring isoform, isoform 1, known as canonical isoform A, was selected.

### 2.1. Compilation of a Dataset of Experimental Protein 3D Models and Identification of Their Valid Subunits

The primary amino acid sequences of CD46, EP, MP, NP, and SP proteins were obtained in fasta format from the UniProt database [19]. The length of these sequences is as follows: 392, 75, 222, 419, and 1273 amino acid residues, respectively. Detailed data can be found in Table S1.

The search for experimental 3D models of human CD46 and EP, MP, NP, and SP proteins of SARS-CoV-2 was performed using the UniProt [19], PDBe [20], and RCSB PDB [21] databases. A total of 281 3D models were found, including 6 models of CD46, 1 model of EP, 21 models of NP, and 253 models of SP. However, no experimental 3D models were found for MP. The PDB codes for all the identified models are listed in Table S2.

None of the identified experimental 3D models contain the complete resolved amino acid sequence of the respective proteins. In some models, the length of the resolved segment is very short (minimum of 9 amino acids in the case of NP). Many 3D models consist of several resolved chains of varying lengths, and some of them are incomplete, composed of multiple fragments.

Taking these facts into consideration, a detailed analysis of the identified 3D models was performed, focusing on selecting adequate isolated 3D subunits in the form of complete resolved fragments for each of the five proteins.

During the assessment of individual subunits in the experimental 3D models, the following criteria were applied:

(1) Substantial length of the modeled amino acid sequence.
(2) Completeness of resolution: the resolved fragmentary subunit should contain minimal gaps in the form of individual non-resolved amino acids.
(3) Incremental increase in the overall resolved length of selected subunits: each newly selected adequate fragment should add an additional resolved segment of the considered protein to the previously identified adequate fragments.

In the six identified experimental 3D models of CD46 protein, 25 adequate fragmentary subunits were identified, with their characteristics provided in Table S3. The total resolved length of the selected 3D subunits of human CD46 protein amounted to 252 amino acids, accounting for 64.3% of the full sequence.

For the EP protein, only one experimental 3D model was found, and it yielded 5 adequate fragmentary subunits, whose characteristics are listed in Table S4. The combined resolved length of the selected 3D subunits of EP protein from SARS-CoV-2 was 31 amino acids, representing 41.3% of the full sequence.

Upon analyzing 21 experimental 3D models of NP protein, 43 adequate fragmentary subunits were identified, with their characteristics provided in Table S5. The cumulative resolved sequence of the selected 3D subunits of NP protein from SARS-CoV-2 covered amino acids 4–180 and 247–364, with a total length of 295 amino acids, equivalent to 70.4% of the full sequence.

For the SP protein, the complete virus protein is a trimer composed of identical subunits. Out of 253 experimental 3D models of SP, we considered the most fully resolved trimeric models, where the configuration was determined for the pure protein without any other associated protein components. Consequently, five 3D models of SP with a well-defined configuration, closely resembling the native structure, were chosen for a further analysis. Characteristics of the adequate fragmentary subunits of SP protein are presented in Table S6. The overall resolved length of the selected 3D subunits of SP protein from SARS-CoV-2 amounted to 1149 amino acids, representing 90.3% of the full sequence.

Validity of adequate isolated subunits: A total of 88 adequate isolated subunits from the experimental 3D models of CD46, EP, NP, and SP proteins were identified, comprising 25, 5, 43, and 15 subunits, respectively.

To assess their validity, the QMEAN-v4.2.0 program [22] was employed. This program evaluates the quality of protein 3D models using the QMEAN4 scoring function, which reflects the degree of native-like structural features observed in the analyzed 3D model. It also characterizes the probability of the 3D model’s quality being comparable to that of a high-resolution experimental 3D construct. The QMEAN algorithm computes and combines both global (for the overall 3D configuration) and local (for individual amino acid residue configurations) quality scores of the tested 3D model. The QMEAN4 function is normalized based on the number of interactions, independent of protein size, and transformed into Z-scores. Accordingly, by definition, sufficiently high-quality 3D models fulfill the condition of statistical significance, |QMEAN4| ≤ 2.

The results of the QMEAN-v4.2.0 calculations for the validation of adequate isolated subunits in the experimental 3D models of CD46, EP, NP, and SP proteins are presented in Tables S7–S10, respectively.

When selecting valid subunits from the experimental 3D models of target proteins, the following criteria were applied:

(1) Subunits with |QMEAN4| > 2 were excluded from consideration.
(2) Among the subunits with |QMEAN4| ≤ 2, priority was given to the longest subunits.
(3) Valid subunits with |QMEAN4| ≤ 2 were selected in a way that increased the overall length of the resolved sequence.
(4) Among multiple subunits of comparable length representing the 3D structure of the same region, two to three subunits with the lowest |QMEAN4| values were chosen.

As a result, to accurately represent the experimental 3D structure of CD46, EP, NP, and SP proteins, 4, 1, 6, and 12 valid subunits were selected, respectively. The characteristics of these selected subunits are presented in Tables S11–S13.

### 2.2. Homology Modeling and Identification of Optimal 3D Models for a Set of Full-Length Protein Structures

Homology modeling of the complete 3D structures of CD46 and EP, MP, NP, and SP was carried out using the I-TASSER-v5.1 program [23]. This program has been recognized as the leading method for 3D protein homology modeling among 215 participants in the CASP project’s 8 series of tests from CASP7 to CASP14, spanning the years 2006 to 2020 [24].

The program employs a hierarchical approach to construct 3D protein models from their amino acid sequences through several stages:

(1) Selection of template proteins from the Protein Data Bank (PDB) based on sequence similarity to the target protein, ensuring these templates have experimentally established 3D structures with mutually similar folds.
(2) Identification of a set of homologous 3D fragments based on the selected templates.
(3) Creation of numerous full-size 3D models of the target protein by assembling the identified homologous 3D fragments and performing subsequent rotations and translations using the Monte Carlo method.
(4) Clustering of the ensemble of 3D structures obtained through simulation using k-means clustering based on free energy.
(5) Determining the most populated cluster, calculating the coordinates of its center, and constructing a 3D structure of the cluster centroid.
(6) Rebuilding a diverse set of full-size 3D models of the target protein by repeating steps 1–3 in relation to the 3D structure of the cluster centroid.
(7) Selection of 3D models with the lowest energy.
(8) Constructing full-atomic 3D models based on the selected full-size 3D models through the optimization of potential hydrogen bond networks.

The I-TASSER-v5.1 program offers a significant advantage by utilizing a double iteration approach in simulating full-size 3D models, leading to the generation of full-atomic 3D models of the target protein. The reliability of the generated 3D models is assessed using the C-score, which takes into account the similarity with template proteins and the convergence parameters of the final model’s 3D structures. A higher C-score indicates a higher level of reliability for the 3D model.

For CD46, EP, MP, NP, and SP proteins, a total of five 3D models for each protein were constructed using the I-TASSER v5.1 program. Additionally, for EP, MP, NP, and SP proteins, a sixth 3D model was built using the D-I-TASSER/C-I-TASSER approach, which is based on deep learning artificial neural networks [25]. The reliability indicators for the generated full-size full-atomic 3D models are provided in Table S14.

Validity assessment: The validity assessment of the homology-based full-atom 3D models of CD46 proteins, EP, NP, and SP was performed using a consensus comparison with valid experimentally resolved 3D subunits of the corresponding proteins. The evaluation involved four similarity comparison programs: DaliLite-v5.3 [26], PDBeFold-v2.58 [27], TM-score-v19.8.22 [28], and FATKAT-v2.0 [29]. The following algorithm was used:

(1) Comparison of full-size 3D models with valid 3D subunits: Each of the 23 homology-based full-atom 3D models was compared with each valid experimentally resolved 3D subunit of the corresponding protein using the four similarity comparison programs. For each pair of comparisons, similarity scores were obtained.
(2) Calculation of rank scores: all similarity index values were converted into rank scores for each protein, and then the average RankMean ranks were calculated.
(3) Consensus estimate of quality: the consensus estimate of the quality of a specific full-size 3D model of a given protein, RankModel, was calculated as the average value of all RankMean indicators.

The application of the consensus approach and rank-based non-parametric statistics provided accurate context-independent integral estimates of the 3D similarity of protein models. The results of the validity assessment for the homology-built full-size 3D models of CD46, EP, NP, and SP are presented in Table S15. Since no experimental 3D models were found for MP, the validity of full-size 3D models constructed from homology was evaluated using the QMEAN-v4.2.0 program [22], and the results are shown in Table S16.

Based on the integrated RankModel score from Table S15, the four best homology-built full-size full-atom 3D models of CD46, EP, NP, and SP were determined. The best homology-built full-size full-atomic 3D model of MP was determined according to the value of the QMEAN4 scoring function from Table S16. The top models in Tables S15 and S16 are highlighted in green, indicating their high reliability and quality among the evaluated models. These top models can serve as reliable starting points for further analyses and investigations related to protein function, interactions, and dynamics.

### 2.3. Generation of Multiple 3D Models of Dimeric Complexes between CD46 Protein and EP, MP, NP, and SP Proteins and Identification of the Most Optimal Conformations

Diheteromeric complexes of the human CD46 protein with SARS-CoV-2 EP, MP, NP, and SP proteins were constructed using several programs, including Cluspro2 [30], GRAMM1 [31], Hex8 [32], SwarmDock [33], and ZDOCK3 [34]. Cluspro2, GRAMM1, Hex8, and ZDOCK3 performed simulations in the hard simulation mode, keeping the initial conformations of the interacting proteins unchanged. On the other hand, SwarmDock conducted simulations in the flexible simulation mode, allowing for changes in the conformations of the interacting proteins.

Modeling of protein complexes in all five programs was carried out in two modes. In the first mode, CD46 was treated as the receptor, and held in a fixed position in space, while EP, MP, NP, and SP proteins were considered as ligands, free to move around CD46 by rotation or movement. In the second mode, the roles were reversed, with EP, MP, NP, and SP acting as the receptors, and CD46 as the ligand.

Cluspro2 [Desta 2020] employed a scoring function to calculate the formation energy of protein–protein complexes, based on interaction networks with one amino acid per cell for 70,000 positions of the ligand. The program selected 1000 positions with the minimum energy, which were then clustered based on the number of amino acids of the receptor, with a distance to the ligand of ≤9 Å. Clusters were ranked by population, and the first 10 were chosen to represent the optimal conformation of the protein–protein complex with the lowest values of formation energy.

In summary, the Cluspro2 program utilized 10 variants characterized by the lowest estimates of free energy from clusters containing the largest number of closely spaced pairs of amino acids to determine the optimal conformations of the protein–protein complex.

GRAMM1 [31] identifies the optimal conformer by smoothing the intermolecular energy function through changes in atomic–atomic potentials. Among 4000 conformations, 300 variants with the lowest energy are selected and subjected to re-optimization, with the choice of one that is energetically the most favorable.

Thus, the GRAMM1 program reveals one of the most optimal conformations of the protein–protein complex, characterized by the minimum value of its intermolecular energy.

Hex8 [32] specifies the surface of each protein as a two-dimensional grid, with parameters of points represented by polar coordinates, steric van der Waals energy, and electrostatic charges. The description of this surface is a three-dimensional expansion of orthogonal spherical polar basis functions. Interaction energy of two proteins is calculated as a scoring function based on the interaction of two solids, obtained by superposition of pairs of parametric three-dimensional functions. The energy is minimized by achieving maximum correlation between the three-dimensional functions forming the scoring function. The parameter values obtained through this optimization correspond to the most favorable conformation of the protein–protein complex.

Thus, in the Hex8 program, one of the most optimal conformations of the protein–protein complex is revealed, which is characterized by the minimum value of the interaction energy.

SwarmDock [33] is based on the Particle Swarm Optimization (PSO) method. The program determines a set of points (amino acids) for both the receptor and ligand. A set of approximately 120 initial positions of the ligand is created, and evenly distributed around the receptor. The PSO search vector, which includes the coordinates of the center of mass and the orientation by the quaternion of the ligand, is optimized to perform the emulation. The objective function to be minimized is defined by the distance-dependent DComplex potential of a pair of points [35].

Optimization in SwarmDock is carried out using a hybrid algorithm that combines PSO (Particle Swarm Optimization) and local search. After each PSO iteration, the element with the lowest energy is subjected to local optimization. This entire process is repeated four times for each of the 120 initial positions. The best structures are then repeatedly minimized in the CHARMM force field, ranked using the centroid potential [35], and subjected to hierarchical clustering based on the number of contacts between the receptor and the ligand. From the first 10 clusters, one structure with the lowest energy is selected.

Thus, in the SwarmDock program, the determination of the optimal conformations of the protein–protein complex is based on the selection of 10 variants characterized by the lowest estimates of the energy of the complex from 10 clusters with the highest scoring estimates of their conformations.

ZDOCK3 [34] defines an initial grid with a step of 1.2 Ǻ, including both receptor and ligand molecules. The ligand and receptor can move and rotate, resulting in the formation of 54,000 possible positions relative to each other. For each position, an energy scoring function is calculated, which includes van der Waals, electrostatic, and IFACE desolvation contact potentials [36]. From these configurations, the 2000 best ones are selected and ranked based on the number of contacts between the receptor and the ligand for amino acids with a distance of ≤6 Ǻ. The scoring functions then select the top 10 positions based on the largest number of contacts and the largest value.

Thus, in the ZDOCK3 program, the determination of the optimal conformations of the protein–protein complex is based on the choice of 10 variants characterized by the largest number of close amino acid contacts and the largest values of the interaction energy scoring function.

The utilization of five diverse programs, each employing different methods for constructing initial conformations, optimization techniques, and evaluation algorithms, allowed for the generation of a comprehensive set of 3D models of diheteromeric complexes involving the human CD46 protein with SARS-CoV-2 EP, MP, NP, and SP proteins, optimizing their parameters.

In total, approximately 1,108,000 configurations were processed during 40 calculation cycles, resulting in 28,160 promising configurations that were further optimized. From these, 238 optimal 3D models of protein–protein complexes were selected, with 119 models featuring CD46 as the receptor and EP, MP, NP, and SP proteins as ligands, and vice versa in the other 119 models, with CD46 acting as the ligand and EP, MP, NP, and SP proteins as the receptors.

Among the 238 identified optimal 3D models, based on scoring scores, the 40 most suitable ones were selected, with two models chosen from each program. Among these selected models, in 20 of them, CD46 acted as the receptor, and EP, MP, NP, and SP proteins served as ligands, while in the other 20 models, CD46 functioned as the ligand, and EP, MP, NP, and SP proteins acted as the receptors. The energy characteristics of these complexes are provided in Table S17.

### 2.4. Analysis of Binding Regions in Adequate 3D Models of Dimeric Protein–Protein Complexes, and Determination of Key Binding Amino Acids in Them

The analysis of the binding regions and identification of key binding amino acids were performed for the 40 most adequate 3D models of human CD46 protein–protein complexes with SARS-CoV-2 EP, MP, NP, and SP proteins using the LigPlotPlus-v1.4.5 program [37].

In each of the four groups of 3D models of protein–protein complexes (CD46↔EP, CD46↔MP, CD46↔NP, and CD46↔SP), comprising the ten most adequate models per group, lists of binding amino acids were generated. This process resulted in eight complete lists of binding amino acids, one list for each protein in each type of protein–protein complex.

The formation of these lists followed a specific algorithm:

(1) For each of the two protein subunits in each of the ten most adequate 3D models of each protein–protein complex type, 80 lists of binding amino acids were created, along with their positions in the primary sequence.
(2) The resulting ten lists for each protein subunit in each protein–protein complex type were combined, and the number of occurrences of each binding amino acid in all ten 3D models was calculated.
(3) The significance metric of a binding amino acid in a given protein subunit of a specific protein–protein complex type was determined based on the number of occurrences in the ten most adequate 3D models.

In the study, a total of 1124 binding amino acids were identified in the best 40 adequate 3D models of CD46 protein–protein complexes. The full lists of these binding amino acids can be found in Tables S18–S21.

The most common binding amino acids observed in protein–protein complexes CD46 ↔ EP, CD46 ↔ MP, CD46 ↔ NP, and CD46 ↔ SP were selected as the key binding amino acids. These key binding amino acids are summarized in Table S22.

The presence of key binding amino acids within the putative regions of the protein–protein complexes was determined for each of the 20 models in each group of CD46 ↔ EP, CD46 ↔ MP, CD46 ↔ NP, and CD46 ↔ SP protein–protein complexes. This analysis allowed us to evaluate the contribution of key binding amino acids to the interactions within each specific 3D model.

Furthermore, we calculated the number of key binding amino acids among the binding amino acids in the proposed protein–protein interaction region for each of the 40 most adequate 3D models of protein–protein complexes. Models with a higher number of key binding amino acids were considered to have more qualitative and significant interactions.

In conclusion, a total of 187 key binding amino acids were identified in the top 40 adequate 3D models of CD46 protein–protein complexes. These key binding amino acids are crucial for understanding the molecular mechanisms and functional implications of these protein–protein interactions.

### 2.5. Selection of the Best 3D Models of Diheteromeric Complexes of the CD46 Protein with EP, MP, NP, and SP Proteins and the Identification of Their Interaction Areas

For each of the 20 pairs of the most adequate 3D models obtained using each of the five programs (ClusPro2, GRAMM1, Hex8, SwarmDock, ZDock3), the total numbers of key binding amino acids found in individual subunits were determined for both complex configurations: CD46 (receptor)_EP MP NP SP (ligand) and EP MP NP SP (receptor)_CD46 (ligand).

Based on these characteristics, percentages of key binding amino acids were calculated for the CD46 subunits in both models, the SARS-CoV-2 protein subunits in both models, the CD46 (receptor)_EP MP NP SP (ligand) complex, the EP MP NP SP (receptor)_CD46 (ligand) complex, and both dimeric complexes.

Using the combination of these percentages, we selected one best pair of 3D models of protein–protein complexes CD46 ↔ EP, CD46 ↔ MP, CD46 ↔ NP, and CD46 ↔ SP. Interestingly, in the selected pairs, the CD46 (receptor)_EP MP NP SP (ligand) and EP MP NP SP (receptor)_CD46 (ligand) models did not significantly differ from each other in terms of their qualitative characteristics. Therefore, we adopted the CD46 (receptor)_EP MP NP SP (ligand) complex model as the best valid model in all four cases.

The results of assessing the quality of models and choosing the best valid 3D model are presented in Tables S23–S26.

Next, the areas of interaction were identified in two stages. Firstly, for each best valid 3D model, lists of key binding amino acids in the subunits of the CD46 protein and SARS-CoV-2 proteins were compiled (Table S27).

Subsequently, at the second stage, each key binding amino acid in one subunit was paired with the spatially most closely located amino acid residue from the other subunit.

By analyzing pairs of key and spatially complementary binding amino acids for each best valid 3D model of the CD46 ↔ EP, CD46 ↔ MP, CD46 ↔ NP, and CD46 ↔ SP complexes, we were able to adequately represent the most probable areas of interaction between the studied protein subunits. This comprehensive approach provides valuable insights into the molecular basis of these protein–protein interactions.

## 3. Results

A total of 281 experimental 3D models (Table S2) were generated for CD46 and EP, MP, NP, and SP proteins, from which 88 isolated subunits were deemed adequate (Tables S3–S10). These subunits were evaluated for their validity using the QMEAN-v4.2.0 program, resulting in the identification of 23 valid subunits (Tables S11–S13). The corresponding structures are provided in the Supplementary Data in pdb format.

Furthermore, 23 full-sized full-atom 3D models of CD46, EP, NP, and SP (Table S14) were constructed using homology-based approaches. To assess their validity, a consensus rank method based on 3D structural similarity with the experimental valid subunits (Table S15) and the QMEAN-v4.2.0 program (Table S16) was employed. Consequently, the five best homology-built full-size full-atom 3D models of CD46, EP, MP, NP, and SP were identified, and their characteristics are detailed in Table 1.

When calculating the average consensus estimates of the validity of the RankModel, the values of the similarity indicators were ranked from the best to the worst, so the best models correspond to the minimum values of the RankModel. For MP, no experimental 3D models were found, so the QMEAN4 scoring function served as an assessment of validity, according to which the best model corresponds to the maximum value of QMEAN4.

The structures of the best full-size 3D models of CD46, EP, MP, NP, and SP, in pdb format, are given in the Supplementary Data.

During the process of constructing 3D models of CD46 dimeric complexes with EP, MP, NP, and SP, in 40 cycles of calculations, approximately 1,108,000 configurations were processed, out of which 28,160 configurations were optimized. From these, 238 optimal 3D models were selected, and then the 40 most adequate models were chosen based on the values of scoring estimates (Table S17).

In these complexes, a total of 1124 binding amino acids were identified (Tables S18–S21), out of which 187 most frequently occurring ones were accepted as key binding amino acids (Table S22).

Generally, the more interacting amino acids, the stronger the protein–protein complex will be. The occurrence of key binding amino acids in all 40 adequate complexes was calculated (Tables S23–S26), and based on the percentage values of these indicators, one best 3D model for each of the CD46 ↔ EP, CD46 ↔ MP, CD46 ↔ NP, and CD46 ↔ SP complexes was selected, the characteristics of which are given in Table 2.

The structures of the best 3D models of CD46 diheteromeric complexes with EP, MP, NP, and SP in pdb format are provided in the Supplementary Data.

For each best valid 3D model of the complexes, pairs of key and spatially complementary binding amino acids were determined, and these are shown in Table 3.

**Figure 1:**
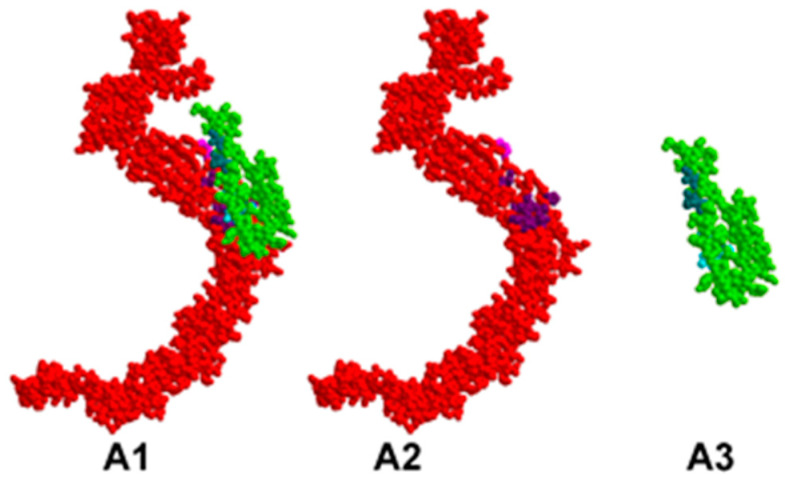
Valid 3D model of human CD46 protein–protein complex and SARS-CoV-2 EP.

**Figure 2:**
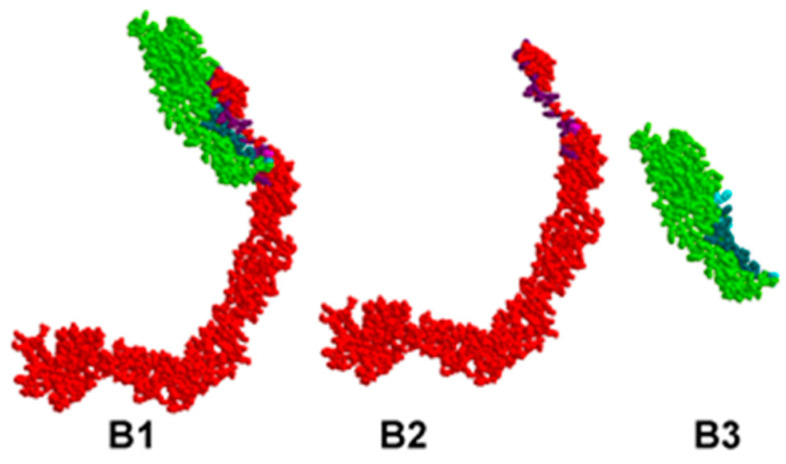
Valid 3D model of the protein–protein complex of human CD46 and SARS-CoV-2 MP.

**Figure 3:**
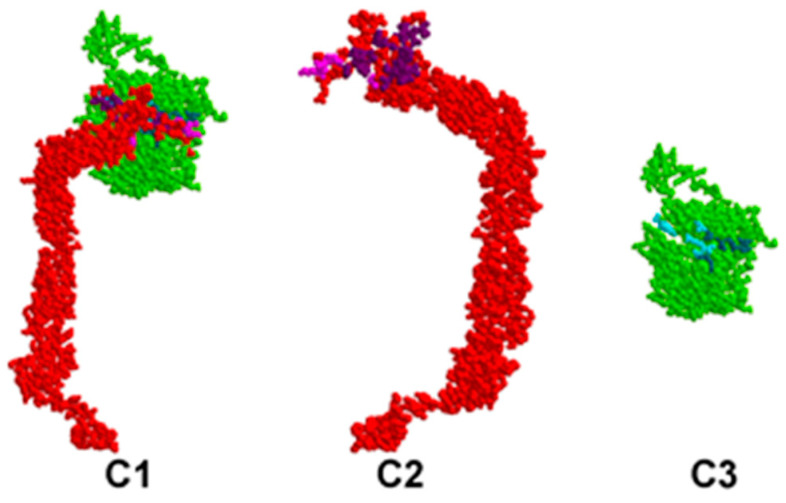
Valid 3D model of human CD46 protein–protein complex and SARS-CoV-2 NP.

**Figure 4:**
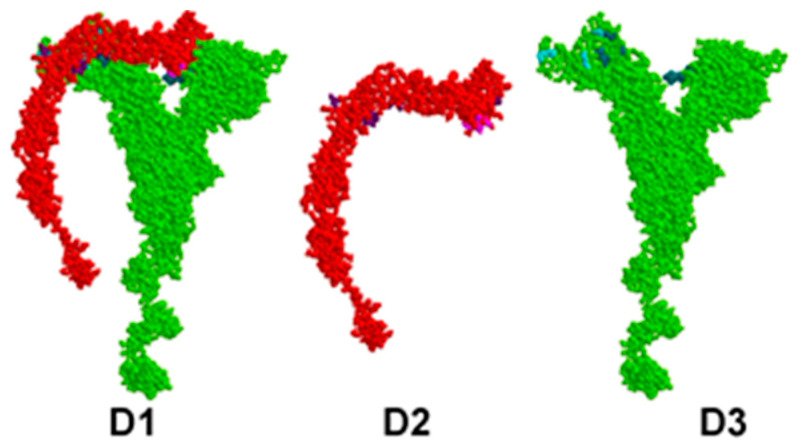
Valid 3D model of human CD46 protein–protein complex and SARS-CoV-2 SP.

The identified pairs of binding amino acids allow for a proper representation of the most probable interaction areas between CD46 and EP, MP, NP, and SP proteins. These interactions are visually presented in Figure 1, Figure 2, Figure 3 and Figure 4.

In all figures, the protein–protein complex is labeled as A1–D1, with CD46 represented as A2–D2, and the second protein (EP, MP, NP, SP) represented as A3–D3.

In each figure, CD46 is highlighted in red, its binding amino acids are depicted in light purple, and key amino acids are shown in dark purple. The SARS-CoV-2 protein is highlighted in green; its binding amino acids are depicted in turquoise, and key amino acids are shown in dark turquoise.

Based on the number of key binding amino acids (NCD46_Prot, as presented in Table 2), the complexes are ranked in the following order: CD46_MP ≈ CD46_NP > CD46_SP >> CD46_EP. Additionally, according to the number of pairs of binding amino acids (NPares, as indicated in Table 3), the complexes are organized in the sequence CD46_NP > CD46_MP = CD46_SP >> CD46_EP.

This suggests that the SARS-CoV-2 NP protein exhibits the most intense interaction with human CD46, followed by the MP protein in terms of interaction intensity, with the S protein in third place, and the EP envelope protein showing the least intensive interaction with CD46.

These findings support the hypothesis that, during nonspecific incorporation into the lipid membrane of a human cell, the configuration of SARS-CoV-2 can significantly change, enabling interactions with other structural proteins of the virus, apart from the S protein. As a result, the S protein loses its leading role as the initiator of SARS-CoV-2 infection. The supposedly stronger binding of CD46 with circulating NP and MP proteins in the blood may potentially exert an influence on the immune system.

This hypothesis opens up a novel avenue for understanding the virus’s capability to infect cells lacking ACE2 expression [2,3,4,5]. CD46, ubiquitously expressed and already known to interact with various pathogens, emerges as a viable putative receptor [8,9,10]. If the hypothesis is confirmed, it may explain a broader range of cellular targets for the virus and might contribute to understanding the severity and complications of the disease. Investigating this interaction can guide the development of new therapeutic strategies such as competitive inhibitors that might block the NP-CD46 or MP-CD46 interaction.

CD46 is known for its role in immune regulation [38,39], and its interaction with circulating NP and MP proteins may have unknown implications for the immune response to SARS-CoV-2. Such interactions could potentially result in immunomodulatory effects, perhaps tilting the immune response toward a proinflammatory or immunosuppressive state. Given the broad spectrum of COVID-19 symptoms and severities, understanding this mechanism could help explain why some cases progress to severe or fatal stages.

Experimental verification is essential for substantiating these hypotheses. For the first, in vitro assays can be designed to test the binding affinity of these proteins to CD46 and to determine whether this binding affects viral entry into cells. For the second, experiments can assess which downstream signaling pathways are activated or inhibited when NP and MP interact with CD46. Subsequently, animal models and clinical trials may be used to validate these mechanisms and their relevance to disease progression and severity.

## 4. Conclusions

In conclusion, the hypothesis presents a multifaceted mechanism through which CD46 may be implicated in both the entry and pathogenesis of SARS-CoV-2, thus opening new avenues for targeted therapeutic strategies. Further experimental research is essential for verifying these proposed mechanisms.

## 5. Study Limitations

In conducting our investigation, it is important to recognize several limitations that need careful consideration. First, when using the docking method to screen low-molecular-weight ligands, there is a risk of type 2 errors: incorrectly identifying inactive compounds as active. This issue, as noted by Ceron-Carrasco (2022) [40], is particularly relevant in the context of SARS-CoV-2 research and underscores the necessity for strong validation protocols. Additionally, the accuracy of docking, as detailed by Chen (2015), varies significantly, ranging from 0% to as high as 92.66% [41]. Such variability calls for a cautious interpretation of our results and highlights the need for continual improvements in methodology to reduce potential errors.

A notable limitation of our study is the absence of molecular dynamics post-docking, as suggested by Biswas (2022) [42]. This was due to resource and scope constraints during our research. We intend to include molecular dynamics in future studies to gain a more thorough understanding of the stability of protein–protein interactions. However, it is crucial to understand that molecular dynamics simulations, despite their potential, are based on minimizing interaction energy within a protein–ligand complex. This approach is similar in principle to docking, as explained by Lazim (2020) [43]. Therefore, there is a risk of converging prematurely on a local energy minimum rather than the more desirable global minimum, presenting a challenge in these methods [43].

In summary, while our study offers valuable insights into ligand screening, these limitations highlight the ongoing need for research and methodological advancements. Such efforts are essential to enhance the accuracy and reliability of our findings in molecular docking and dynamics.

## Figures and Tables

**Table 1:** Validity scores of the best full-size 3D models of CD46, EP, MP, NP, and SP based on homology.

Protein	Model Code	RankModel	Max RankModel
CD46	Model1	8.02	15.08
EP	QHD43418	2.10	7.80
NP	QHD43423	6.36	24.89
SP	Model5	31.34	40.45
MP	QHD43419	QMEAN4 = −5.79	QMEAN4 = −16.07

**Table 2:** Number of key binding amino acids in the best 3D models of CD46 complexes with EP, MP, NP, and SP.

Model Code ^1^	N_All_^2^	N_CD46_Prot_^3^	Pr_KBAA_^4^
CD46_EP_GRAMM1	26	15	57.7
CD46_MP_ZDock3	37	29	78.4
CD46_NP_ZDock3	62	28	45.2
CD46_SP_ZDock3	44	23	52.3

^1^ Program name included. ^2^ Number of all found. ^3^ The number in the complex. ^4^ Percent in the complex.

**Table 3:** Key and complementary amino-acid-binding interaction regions in the best valid 3D models of CD46 complexes with EP, MP, NP, and SP.

Model Code	Amino Acids ^1^	NPares ^2^
CD46_EP_GRAMM1	**THR188**–ILE33, **TYR189**–ALA32, **SER190**–**VAL29**, **CYS191**–**VAL29**, **PRO193**–VAL75, **PHE200**–VAL25, **LEU202**–**VAL29**, **GLU205**–**VAL29**, **SER206**–ALA32, **THR207**–THR35, LYS244–**ILE13**, LYS244–**VAL17**, **TYR247**–**PHE20**	13
CD46_MP_ZDock3	**PHE11**–PHE100, **PRO12**–PHE100, **TRP14**–**PHE96**, **PHE16**–**TRP92**, **PRO17**–LEU93, **LEU20**–**TRP92**, **MET24**–**LEU56**, **MET24**–**PRO59**, **VAL25**–**LEU57**, **VAL25**–**VAL60**, **LEU26**–**LEU62**, **LEU27**–**ALA63**, **TYR29**–**VAL66**, **TYR29**–**LEU67, PHE31**–**MET1**, **PHE31**–**VAL70**, SER32–**ASP3**, **GLU36**–SER4, **ASN83**–**TYR71**, **THR85**–**TYR71**	20
CD46_NP_ZDock3	PRO334–**SER21**, **ILE338**–**PHE17**, **SER341**–THR16, **LEU342**–ILE15, **ASP343**–ILE15, **VAL344**–ARG14, **TRP345**–ARG14, **VAL346**–ILE15, **ILE347**–THR16, **VAL351**–GLN242, **ALA353**–GLN241, **ILE354**–GLN241, **VAL355**–GLN240, **ALA359**–GLN241, **VAL360**–GLN241, **TYR366**–**LEU219**, **TYR368**–**LEU219**, ARG372–**ALA211**, ARG372–**GLY212**, LYS373–**ASN213**, LYS374–**ASP216**, **TYR378**–**LEU223**, **THR380**–**LEU223**, **THR383**–LEU227, **HIS384**–LEU227	25
CD46_SP_ZDock3	**GLU177**–ASN487, **VAL178**–PHE486, **PRO199**–ASN501, **GLU205**–LYS417, **ARG218**–GLY416, **SER240**–**SER375**, TYR247–**ASP405**, **TYR248**–**ARG408, TYR248**–**GLY502**, **LYS249**–**VAL503**, **LYS293**–THR114, **PRO295**–LYS113, SER305–**ASP198**, THR306–**GLY199**, SER315–**PHE2**, ASN327–**MET1**, TYR328–**VAL6**, TYR331–**PHE4**, TYR331–**LEU5**, LYS333–**VAL3**	20

^1^ Key binding amino acids are in bold type, complementary amino acids are in regular type. ^2^ Number of pairs of binding amino acids.

## Data Availability

The data presented in this study are available on request from the corresponding author and in supplementary files.

## Supplementary Materials

The following supporting information can be downloaded at: https://www.mdpi.com/article/10.3390/v15122297/s1, Table S1: Main characteristics of relevant target proteins; Table S2. Experimental 3D models of relevant target proteins; Table S3. Adequate resolved isolated subunits of experimental 3D models of human CD46 membrane cofactor protein; Table S4. Adequate resolved isolated subunits of experimental 3D models of SARS-CoV-21 envelope protein (EP); Table S5. Adequate resolved isolated subunits of experimental 3D models of SARS-CoV-2 1 nucleocapsid protein (NP); Table S6. Adequate resolved isolated subunits of experimental 3D models of SARS-CoV-2 1 spike protein (SP); Table S7. Validity indicators of adequate resolved isolated subunits from experimental 3D models of human CD46 membrane cofactor protein; Table S8. Validity Indicators of Adequate Resolved Isolated Subunits from Experimental 3D Models of SARS-CoV-21 Envelope Protein (EP); Table S9. Validity Indicators of Adequate Resolved Isolated Subunits from Experimental 3D Models of SARS-CoV-2 Nucleocapsid Protein (NP); Table S10. Validity Indicators of Adequate Resolved Isolated Subunits from Experimental 3D Models of SARS-CoV-2 1 Spike Protein (SP); Table S11. Valid Subunits Representing the Experimental 3D Structure of Human CD46 Membrane Cofactor Protein; Table S12. Valid Subunits Representing the Experimental 3D Structure of SARS-CoV-2 Nucleocapsid Protein (NP); Table S13. Valid Subunits Representing the Experimental 3D Structure of SARS-CoV-2 Spike Protein (SP); Table S14. Reliability of Homology-Based Full 3D Models of Human CD46 Protein and SARS-CoV-2 Envelope Protein (EP), Membrane Protein (MP), Nucleocapsid Protein; Table S15. Ranking of Validity Assessment for Homology-Based Full-Size 3D Models of Human CD46 Protein and SARS-CoV-2 Envelope Protein (EP), Membrane Protein (MP), Nucleocapsid Protein (NP), and Spike Protein (SP); Table S16. Validity Assessment of Homology-Based Full-Size 3D Models of SARS-CoV-2 Membrane Protein (MP); Table S17. Energetic Characteristics of Protein-Protein Complexes of Human CD46 and SARS-CoV-2 Envelope Protein (EP), Membrane Protein (MP), Nucleocapsid Protein (NP), and Spike Protein (SP); Table S18. Full Lists of Binding Amino Acids in the Human CD46 and SARS-CoV-2 Envelope Protein (EP) Protein-Protein Complex; Table S19. Full Lists of Binding Amino Acids in the Human CD46 and SARS-CoV-2 Membrane Protein (MP) Protein-Protein Complex; Table S20. Full Lists of Binding Amino Acids in the Human CD46 and SARS-CoV-2 Nucleocapsid Protein (NP) Protein-Protein Complex; Table S21. Full Lists of Binding Amino Acids in the Human CD46 and SARS-CoV-2 Spike Protein (SP) Protein-Protein Complex; Table S22. Key Binding Amino Acids in the Human CD46 and SARS-CoV-2 Envelope Protein (EP), Membrane Protein (MP), Nucleocapsid Protein (NP), and Spike Protein (SP) Protein-Protein Complexes; Table S23. Quality Assessment of 3D Models of Human CD46 and SARS-CoV-2 Envelope Protein (EP) Protein-Protein Complexes Obtained Using Different Software Tools; Table S24. Quality Assessment of 3D Models of Human CD46 and SARS-CoV-2 Membrane Protein (MP) Protein-Protein Complexes Obtained Using Different Software Tools; Table S25. Quality Assessment of 3D Models of Human CD46 and SARS-CoV-2 Nucleocapsid Protein (NP) Protein-Protein Complexes Obtained Using Different Software Tools; Table S26. Quality Assessment of 3D Models of Human CD46 and SARS-CoV-2 Spike Protein (SP) Protein-Protein Complexes Obtained Using Different Software Tools; Table S27. Key Binding Amino Acids in the Interaction Regions of Subunits in the Best Valid 3D Models of Human CD46 and SARS-CoV-2 Envelope Protein (EP), Membrane Protein (MP), Nucleocapsid Protein (NP), and Spike Protein (SP) Protein-Protein Complexes. The supporting information can be also downloaded at: https://data.mendeley.com/datasets/9s3b5m76n6/1 (access on 16 October 2023).
