# Supplementary material for: Computational Analysis of CD46 Protein Interaction with SARS-CoV-2 Structural Proteins: Elucidating a Putative Viral Entry Mechanism into Human Cells
Source: Viruses. 2023 Nov 23;15(12):2297. doi: 10.3390/v15122297 (PMC10747966; doi:10.3390/v15122297)
Supplement: Supplementary file 1 [file viruses-15-02297-s001.zip › viruses-2705612-supplementary.pdf]

**Table S1.** Main characteristics of relevant target proteins.

| Protein [1] | Gene [2] | Code [3] | Name [4] | Organism | Length [5] |
|---|---|---|---|---|---|
| CD46 | CD46 | P15529 | Membrane cofactor protein | Homo sapiens | 392 |
| EP | E | P0DTC4 | Envelope small membrane protein | SARS-CoV-2 | 75 |
| MP | M | P0DTC5 | Membrane protein | SARS-CoV-2 | 222 |
| NP | N | P0DTC9 | Nucleoprotein | SARS-CoV-2 | 419 |
| SP | S | P0DTC2 | Spike glycoprotein | SARS-CoV-2 | 1273 |

Note.1. Generally accepted protein abbreviation; 2. Gene's nomenclatural designation; 3. Common protein code recommended by UniProt.; 4. Nomenclature name recommended by UniProt.; 5. Length of the primary sequence in amino acid residues.

**Table S2**. Experimental 3D models of relevant target proteins.

| Protein | PDB Code |
|---|---|
| CD46 | 1CKL, 2O39, 3INB, 3L89, 3O8E, 5F08 |
| EP | 7K3G |
| MP | – |
| NP | 6M3M, 6VYO, 6WJI, 6WKP, 6WZO, 6WZQ, 6YI3, 6YUN, 6ZCO, 7ACS, 7ACT, 7C22, 7CDZ, 7CE0, 7DE1, 7KGO, 7KGP, 7KGQ, 7KGR, 7KGT, 7RGS |
| SP | 6LVN, 6LXT, 6LZG, 6MO5, 6M17, 6M1V, 6VSB, 6VW1, 6VXX, 6VYB, 6W41, 6WPS, 6WPT, 6X29, 6X2A, 6X2B, 6X2C, 6X6P, 6X79, 6XC2, 6XC3, 6XC4, 6XC7, 6XCM, 6XCN, 6XDG, 6XE1, 6XEY, 6XF5, 6XF6, 6XKL, 6XKP, 6XKQ, 6XLU, 6XMO, 6XM3, 6XM4, 6XM5, 6XR8, 6XRA, 6XS6, 6YLA, 6YM0, 6YOR, 6YZ5, 6YZ7, 6Y2M, 6Z43, 6Z97, 6ZB4, 6ZB5, 6ZBP, 6ZCZ, 6ZDG, 6ZDH, 6ZER, 6ZFO, 6ZGE, 6ZGG, 6ZGI, 6ZH9, 6ZHD, 6ZLR, 6ZOW, 6ZOX, 6ZOY, 6ZOZ, 6ZP0, 6ZP1, 6ZP2, 6ZP5, 6ZP7, 6ZWV, 6ZXN, 7A25, 7A29, 7A4N, 7A5R, 7A5S, 7A91, 7A92, 7A93, 7A94, 7A95, 7A96, 7A97, 7A98, 7AD1, 7B17, 7BEH, 7BEI, 7BEJ, 7BEK, 7BEL, 7BEM, 7BEN, 7BEO, 7BEP, 7BWJ, 7BYR, 7BZS, 7C01, 7C2L, 7C8D, 7C8J, 7C8V, 7C8W, 7CAB, 7CAC, 7CAH, 7CAI, 7CAK, 7CAN, 7CDI, 7CDJ, 7CH4, 7CH5, 7CHB, 7CHC, 7CHE, 7CHF, 7CHH, 7CJF, 7CM4, 7CN9, 7CT5, 7CWL, 7CWM, 7CWN, 7CWO, 7CWS, 7CWU, 7D2Z, 7D30, 7DCC, 7DCX, 7DD2, 7DD8, 7DDD, 7DDN, 7DF3, 7DF4, 7DK3, 7DK4, 7DK5, 7DK6, 7DK7, 7DMU, 7DPM, 7JJC, 7JJI, 7JJJ, 7JMO, 7JMP, 7JMW, 7JV2, 7JV4, 7JV6, 7JVA, 7JVB, 7JVC, 7JW0, 7JWB, 7JWY, 7JX3, 7JZL, 7JZM, 7JZN, 7JZU, 7K43, 7K45, 7K4N, 7K8M, 7K8S, 7K8T, 7K8U, 7K8V, 7K8W, 7K8X, 7K8Y, 7K8Z, 7K90, 7K9Z, 7KDG, 7KDH, 7KDI, 7KDJ, 7KDK, 7KDL, 7KE4, 7KE6, 7KE7, 7KE8, 7KE9, 7KEA, 7KEB, 7KEC, 7KGJ, 7KGK, 7KJ2, 7KJ3, 7KJ4, 7KJ5, 7KKK, 7KKL, 7KL9, 7KLG, 7KLH, 7KLW, 7KMB, 7KMG, 7KMH, 7KMI, 7KMK, 7KML, 7KMS, 7KMZ, 7KN5, 7KN6, 7KN7, 7KNB, 7KNE, 7KNH, 7KHI, 7KS9, 7KSG, 7KSJ, 7KXK, 7KZB, 7L02, 7L06, 7L09, 7L0N, 7L2C, 7L3N, 7L5B, 7L7F, 7L7K, 7LCN, 7LD1, 7LOP, 7ND3, 7ND4, 7ND5, 7ND6, 7ND7, 7ND8, 7ND9, 7NDA, 7NDB, 7NDC, 7NDD, 7NEH |

**Table S3**. Adequate resolved isolated subunits of experimental 3D models of human CD46 membrane cofactor protein.

| PDB Code | Chain [1] | Designation [2] | Length [3] | Range [4] |
|---|---|---|---|---|
| 1CKL | A | CD46_1CKL-A | 126 | 35-160 |
| 1CKL | B | CD46_1CKL-B | 126 | 35-160 |
| 1CKL | C | CD46_1CKL-C | 126 | 35-160 |
| 1CKL | D | CD46_1CKL-D | 126 | 35-160 |
| 1CKL | E | CD46_1CKL-E | 126 | 35-160 |
| 1CKL | F | CD46_1CKL-F | 126 | 35-160 |
| 2O39 | C | CD46_2O39-C | 126 | 35-160 |
| 2O39 | D | CD46_2O39-D | 126 | 35-160 |
| 3INB | C | CD46_3INB-C | 126 | 35-160 |
| 3INB | D | CD46_3INB-D | 126 | 35-160 |
| 3L89 | M | CD46_3L89-M | 126 | 35-160 |
| 3L89 | N | CD46_3L89-N | 126 | 35-160 |
| 3L89 | O | CD46_3L89-O | 126 | 35-160 |
| 3L89 | P | CD46_3L89-P | 126 | 35-160 |
| 3L89 | Q | CD46_3L89-Q | 125 | 35-160 |
| 3L89 | R | CD46_3L89-R | 126 | 35-160 |
| 3L89 | S | CD46_3L89-S | 126 | 35-160 |
| 3L89 | T | CD46_3L89-T | 124 | 35-160 |
| 3L89 | U | CD46_3L89-U | 126 | 35-160 |
| 3L89 | V | CD46_3L89-V | 126 | 35-160 |
| 3L89 | W | CD46_3L89-W | 125 | 35-160 |
| 3L89 | X | CD46_3L89-X | 126 | 35-160 |
| 3O8E | B | CD46_3O8E-B | 252 | 35-286 |
| 3O8E | D | CD46_3O8E-D | 248 | 35-286 |
| 5FO8 | C | CD46_5FO8-C | 129 | 158-286 |

Note. 1. According to their designations in the original 3D model. 2. Designation of the isolated resolved subunit and the name of the pdb file containing this subunit. 3. Length of the isolated resolved subunit in amino acid residues. 4. The starting and ending numbers of the amino acids in the continuous resolved sequence.

**Table S4**. Adequate resolved isolated subunits of experimental 3D models of SARS-CoV-2[1] envelope protein (EP).

| PDB Code | Chain | Designation | Length | Range |
|---|---|---|---|---|
| 7K3G | A | EP_7K3G-A | 31 | 8-38 |
| 7K3G | B | EP_7K3G-B | 31 | 8-38 |
| 7K3G | C | EP_7K3G-C | 31 | 8-38 |
| 7K3G | D | EP_7K3G-D | 31 | 8-38 |
| 7K3G | E | EP_7K3G-E | 31 | 8-38 |

Note. 1. The column designations correspond to Table S3.

**Table S5**. Adequate resolved isolated subunits of experimental 3D models of SARS-CoV-2 [1] nucleocapsid protein (NP).

| PDB Code | Chain | Designation | Length | Range |
|---|---|---|---|---|
| 6M3M | A | NP_6M3M-A_102-174 | 73 | 102-174 |
| 6M3M | A | NP_6M3M-A_49-96 | 48 | 49-96 |
| 6M3M | B | NP_6M3M-B_48-97 | 50 | 48-97 |
| 6M3M | B | NP_6M3M-B_99-174 | 76 | 99-174 |
| 6M3M | C | NP_6M3M-C_47-96 | 50 | 47-96 |
| 6M3M | C | NP_6M3M-C_98-173 | 76 | 98-173 |
| 6M3M | D | NP_6M3M-D_101-174 | 74 | 101-174 |
| 6M3M | D | NP_6M3M-D_48-99 | 52 | 48-99 |
| 6VYO | D | NP_6YVO-D | 125 | 49-173 |
| 6WJI | A | NP_6WIJ-A | 108 | 257-364 |
| 6WJI | B | NP_6WIJ-B | 108 | 257-364 |
| 6WJI | C | NP_6WIJ-C | 108 | 257-364 |
| 6WJI | D | NP_6WIJ-D | 108 | 257-364 |
| 6WJI | E | NP_6WIJ-E | 108 | 257-364 |
| 6WJI | F | NP_6WIJ-F | 108 | 257-364 |
| 6WZO | A | NP_6WZO-A | 108 | 257-364 |
| 6WZO | B | NP_6WZO-B | 111 | 254-364 |
| 6WZO | C | NP_6WZO-C | 108 | 257-364 |
| 6WZO | D | NP_6WZO-D | 110 | 255-364 |
| 6WZQ | A | NP_6WZQ-A | 114 | 251-364 |
| 6WZQ | B | NP_6WZQ-B | 114 | 251-364 |
| 6WZQ | C | NP_6WZQ-C | 116 | 249-364 |
| 6WZQ | D | NP_6WZQ-D | 115 | 250-364 |
| 6YI3 | A | NP_6YI3-A | 137 | 44-180 |
| 6YUN | A | NP_6YUN-A | 116 | 249-364 |

| | | | | |
|---|---|---|---|---|
| 6YUN | B | NP_6YUN-B | 118 | 247-364 |
| 6ZCO | A | NP_6ZCO-A | 118 | 247-364 |
| 7ACS | A | NP_7ACS-A | 137 | 44-180 |
| 7ACT | A | NP_7ACT-A | 137 | 44-180 |
| 7C22 | A | NP_7C22-A | 109 | 256-364 |
| 7C22 | B | NP_7C22-B | 113 | 252-364 |
| 7C22 | C | NP_7C22-C | 112 | 253-364 |
| 7C22 | D | NP_7C22-D | 108 | 257-364 |
| 7CDZ | A | NP_7CDZ-A | 128 | 4-131 |
| 7CDZ | B | NP_7CDZ-B | 127 | 4-130 |
| 7CDZ | C | NP_7CDZ-C | 126 | 5-130 |
| 7CDZ | D | NP_7CDZ-D | 127 | 5-131 |
| 7CE0 | A | NP_7CE0-A | 110 | 255-364 |
| 7CE0 | B | NP_7CE0-B | 110 | 255-364 |
| 7CE0 | C | NP_7CE0-C | 110 | 255-364 |
| 7CE0 | D | NP_7CE0-D | 110 | 255-364 |
| 7DE1 | A | NP_7DE1-A | 108 | 257-364 |
| 7DE1 | B | NP_7DE1-B | 115 | 250-364 |

Note. 1. The column designations correspond to Table S3.

**Table S6.** Adequate resolved isolated subunits of experimental 3D models of SARS-CoV-2 [1] spike protein (SP).

| PDB Code | Chain | Designation | Length | Range |
|---|---|---|---|---|
| 6XR8 | A | SP_6XR8-A | 1107 | 14-1162 |
| 6XR8 | B | SP_6XR8-B | 1107 | 14-1162 |
| 6XR8 | C | SP_6XR8-C | 1107 | 14-1162 |
| 6ZWV | A | SP_6ZWV-A | 985 | 27-1151 |
| 6ZWV | B | SP_6ZWV-B | 985 | 27-1151 |
| 6ZWV | C | SP_6ZWV-C | 985 | 27-1151 |
| 7DDD | A | SP_7DDD-A | 1088 | 14-1147 |
| 7DDD | B | SP_7DDD-B | 1088 | 14-1147 |
| 7DDD | C | SP_7DDD-C | 1088 | 14-1147 |
| 7JJI | A | SP_7JJI-A | 1109 | 14-1146 |
| 7JJI | B | SP_7JJI-B | 1109 | 14-1146 |
| 7JJI | C | SP_7JJI-C | 1109 | 14-1146 |
| 7L7K | A | SP_7L7K-A | 973 | 27-1146 |
| 7L7K | B | SP_7L7K-B | 973 | 27-1146 |
| 7L7K | C | SP_7L7K-C | 973 | 27-1146 |

Note. 1. Column designations correspond to Table S3.

**Table S7.** Validity indicators of adequate resolved isolated subunits from experimental 3D models of human CD46 membrane cofactor protein.

| Designation [1] | QMEAN4 [2] | Designation [1] | QMEAN4 [2] |
|---|---|---|---|
| CD46_1CKL-A | -4.95 | CD46_3L89-P | -0.47 |
| CD46_1CKL-B | -0.66 | CD46_3L89-Q | -1.37 |
| CD46_1CKL-C | -3.40 | CD46_3L89-R | -0.05 |
| CD46_1CKL-D | -2.09 | CD46_3L89-S | -0.44 |
| CD46_1CKL-E | -1.62 | CD46_3L89-T | -0.37 |
| CD46_1CKL-F | -0.50 | CD46_3L89-U | 0.16 |
| CD46_2O39-C | -1.39 | CD46_3L89-V | -0.68 |
| CD46_2O39-D | -0.86 | CD46_3L89-W | -0.53 |
| CD46_3INB-C | -6.13 | CD46_3L89-X | -0.18 |
| CD46_3INB-D | -6.94 | CD46_3O8E-B | 0.96 |
| CD46_3L89-M | -0.48 | CD46_3O8E-D | 1.42 |
| CD46_3L89-N | -0.73 | CD46_5FO8-C | 0.08 |
| CD46_3L89-O | -0.26 | | |

Note. 1. Designation of the pdb file containing the respective isolated subunit. 2. The validity assessment function for the 3D model [Benkert, 2011].

**Table S8.** Validity Indicators of Adequate Resolved Isolated Subunits from Experimental 3D Models of SARS-CoV-2[1] Envelope Protein (EP).

| Designation | QMEAN4 | Designation | QMEAN4 |
|---|---|---|---|
| EP_7K3G-A | -2.90 | EP_7K3G-D | -2.90 |
| EP_7K3G-B | -2.94 | EP_7K3G-E | -2.90 |
| EP_7K3G-C | -2.87 | | |

Note. 1. The column designations correspond to Table S7.

**Table S9.** Validity Indicators of Adequate Resolved Isolated Subunits from Experimental 3D Models of SARS-CoV-2 Nucleocapsid Protein (NP).

| Designation | QMEAN4 | Designation | QMEAN4 | Designation | QMEAN4 |
|---|---|---|---|---|---|
| NP_6M3M-A_102-174 | 0.17 | NP_6WZO-A | -0.12 | NP_7C22-B | 0.12 |
| NP_6M3M-A_49-96 | -1.40 | NP_6WZO-B | 0.07 | NP_7C22-C | -0.23 |
| NP_6M3M-B_48-97 | -0.71 | NP_6WZO-C | 0.07 | NP_7C22-D | -0.15 |
| NP_6M3M-B_99-174 | -0.17 | NP_6WZO-D | 0.01 | NP_7CDZ-A | 0.94 |
| NP_6M3M-C_47-96 | -1.10 | NP_6WZQ-A | 0.22 | NP_7CDZ-B | 1.57 |
| NP_6M3M-C_98-173 | -0.74 | NP_6WZQ-B | 0.10 | NP_7CDZ-C | 0.83 |
| NP_6M3M-D_101-174 | -0.34 | NP_6WZQ-C | 0.37 | NP_7CDZ-D | 1.27 |

| | | | | | |
|---|---|---|---|---|---|
| NP_6M3M-D_48-99 | -1.20 | NP_6WZQ-D | -0.27 | NP_7CE0-A | 0.11 |
| NP_6YVO-D | 1.19 | NP_6YI3-A | not valid [2] | NP_7CE0-B | 0.20 |
| NP_6WIJ-A | -0.16 | NP_6YUN-A | 0.39 | NP_7CE0-C | 0.37 |
| NP_6WIJ-B | -0.19 | NP_6YUN-B | 0.13 | NP_7CE0-D | 0.26 |
| NP_6WIJ-C | -0.14 | NP_6ZCO-A | -0.04 | NP_7DE1-A | 0.00 |
| NP_6WIJ-D | -0.17 | NP_7ACS-A | not valid [2] | NP_7DE1-B | 0.18 |
| NP_6WIJ-E | 0.04 | NP_7ACT-A | -3.22 | | |
| NP_6WIJ-F | -0.16 | NP_7C22-A | -0.25 | | |

Note. 1. The column designations correspond to Table 7. 2. QMEAN values are exceeding the permissible range,

**Table S10.** Validity Indicators of Adequate Resolved Isolated Subunits from Experimental 3D Models of SARS-CoV-2 [1] Spike Protein (SP).

| Designation | QMEAN4 | Designation | QMEAN4 |
|---|---|---|---|
| SP_6XR8-A | -0.57 | SP_7DDD-C | -1.68 |
| SP_6XR8-B | -0.65 | SP_7JJI-A | 0.87 |
| SP_6XR8-C | -0.72 | SP_7JJI-B | 0.91 |
| SP_6ZWV-A | -1.28 | SP_7JJI-C | 0.91 |
| SP_6ZWV-B | -1.26 | SP_7L7K-A | -2.16 |
| SP_6ZWV-C | -1.20 | SP_7L7K-D | -3.33 |
| SP_7DDD-A | -1.65 | SP_7L7K-C | -2.15 |
| SP_7DDD-B | -1.67 | | |

Note. 1. The column designations correspond to Table S7.

**Table S11.** Valid Subunits Representing the Experimental 3D Structure of Human CD46 Membrane Cofactor Protein.

| Designation | Length | Range | QMEAN4 |
|---|---|---|---|
| CD46_3L89-R | 126 | 35-160 | -0.05 |
| CD46_3O8E-B | 252 | 35-286 | 0.96 |
| CD46_3O8E-D | 248 | 35-286 | 1.42 |
| CD46_5FO8-C | 129 | 158-286 | 0.08 |

**Table S12.** Valid Subunits Representing the Experimental 3D Structure of SARS-CoV-2 Nucleocapsid Protein (NP).

| Designation | Length | Range | QMEAN |
|---|---|---|---|
| NP_6YVO-D | 125 | 49-173 | 1.19 |
| NP_6WZQ-C | 116 | 249-364 | 0.37 |
| NP_6YUN-A | 116 | 249-364 | 0.39 |
| NP_7CDZ-B | 127 | 4-130 | 1.57 |
| NP_7CDZ-D | 127 | 5-131 | 1.27 |
| NP_7CE0-C | 110 | 255-364 | 0.37 |

**Table S13.** Valid Subunits Representing the Experimental 3D Structure of SARS-CoV-2 Spike Protein (SP).

| Designation | Length | Range | QMEAN |
|---|---|---|---|
| SP_6XR8-A | 1107 | 14-1162 | -0.57 |
| SP_6XR8-B | 1107 | 14-1162 | -0.65 |
| SP_6XR8-C | 1107 | 14-1162 | -0.72 |
| SP_6ZWV-A | 985 | 27-1151 | -1.28 |
| SP_6ZWV-B | 985 | 27-1151 | -1.26 |
| SP_6ZWV-C | 985 | 27-1151 | -1.20 |
| SP_7DDD-A | 1088 | 14-1147 | -1.65 |
| SP_7DDD-B | 1088 | 14-1147 | -1.67 |
| SP_7DDD-C | 1088 | 14-1147 | -1.68 |
| SP_7JJI-A | 1109 | 14-1146 | 0.87 |
| SP_7JJI-B | 1109 | 14-1146 | 0.91 |
| SP_7JJI-C | 1109 | 14-1146 | 0.91 |

**Table S14.** Reliability of Homology-Based Full 3D Models of Human CD46 Protein and SARS-CoV-2 Envelope Protein (EP), Membrane Protein (MP), Nucleocapsid Protein

| Protein | C-score for Model No. | | | | |
|---|---|---|---|---|---|
| | 1 | 2 | 3 | 4 | 5 |
| Human CD46 | -0.84 | -3.27 | -2.29 | -3.39 | -3.77 |
| EP SARS-CoV-2 | -0.85 | -1.66 | -1.01 | -1.05 | -3.90 |
| MP SARS-CoV-2 | -3.35 | -3.43 | -4.81 | -5.00 | -4.94 |
| NP SARS-CoV-2 | -1.58 | -1.95 | -3.18 | -2.73 | -3.04 |
| SP SARS-CoV-2 | -2.07 | -2.12 | -2.37 | -2.61 | -2.69 |

**Table S15.** Ranking of Validity Assessment for Homology-Based Full-Size 3D Models of Human CD46 Protein and SARS-CoV-2 Envelope Protein (EP), Membrane Protein (MP), Nucleocapsid Protein (NP), and Spike Protein (SP).

| Experimental Subunit | Rank | | | | | | | | | | | | | |
|---|---|---|---|---|---|---|---|---|---|---|---|---|---|---|
| | **DALI** | | **PDBeFold** | | | | **TM-score** | | | **FATKAT** | | | **Rank$_{Mean}$** | **Rank$_{Model}$** |
| | **Z** | **RSMD** | **Q** | **P** | **Z** | **RSMD** | **RSMD** | **TM** | **p** | **Score** | **RSMD$_{opt}$** | **RSMD$_{chain}$** | | |
| **Human CD46 Membrane Protein** | | | | | | | | | | | | | | |
| **Model1** | | | | | | | | | | | | | | **8.02** |
| CD46_3O8E-B | 3 | 1 | 1 | 3 | 5 | 1 | 7 | 4 | 1 | 3 | 1 | 1 | 2.58 | |
| CD46_3O8E-D | 5 | 2 | 4 | 4 | 7 | 4 | 1 | 13 | 1 | 7 | 4 | 4 | 4.67 | |
| CD46_5FO8-C | 10 | 9 | 9 | 9 | 9 | 9 | 18 | 17 | 1 | 11 | 9 | 9 | 10.00 | |
| CD46_3L89-R | 16 | 13 | 11 | 13 | 13 | 13 | 19 | 14 | 15 | 18 | 18 | 15 | 14.83 | |
| Model2 | | | | | | | | | | | | | | 9.10 |
| CD46_3O8E-B | 4 | 2 | 3 | 1 | 6 | 3 | 9 | 5 | 1 | 2 | 3 | 3 | 3.50 | |
| CD46_3O8E-D | 7 | 2 | 5 | 2 | 8 | 5 | 3 | 18 | 1 | 6 | 5 | 5 | 5.58 | |
| CD46_5FO8-C | 9 | 10 | 18 | 14 | 17 | 18 | 20 | 16 | 10 | 10 | 10 | 10 | 13.50 | |
| CD46_3L89-R | 15 | 16 | 13 | 11 | 12 | 14 | 15 | 3 | 16 | 16 | 19 | 16 | 13.83 | |
| Model3 | | | | | | | | | | | | | | 10.06 |
| CD46_3O8E-B | 5 | 7 | 7 | 5 | 1 | 7 | 11 | 1 | 1 | 4 | 7 | 7 | 5.25 | |
| CD46_3O8E-D | 8 | 7 | 8 | 6 | 2 | 8 | 5 | 20 | 1 | 8 | 8 | 8 | 7.42 | |
| CD46_5FO8-C | 13 | 12 | 19 | 14 | 19 | 19 | 13 | 19 | 12 | 14 | 12 | 12 | 14.83 | |
| CD46_3L89-R | 14 | 13 | 12 | 12 | 11 | 11 | 14 | 6 | 14 | 17 | 16 | 13 | 12.75 | |
| Model4 | | | | | | | | | | | | | | 8.56 |
| CD46_3O8E-B | 1 | 2 | 2 | 7 | 3 | 2 | 10 | 2 | 1 | 1 | 2 | 2 | 2.92 | |
| CD46_3O8E-D | 2 | 2 | 5 | 8 | 4 | 6 | 4 | 12 | 1 | 5 | 5 | 5 | 4.92 | |
| CD46_5FO8-C | 10 | 10 | 20 | 14 | 17 | 20 | 17 | 15 | 10 | 12 | 11 | 11 | 13.92 | |
| CD46_3L89-R | 12 | 13 | 10 | 10 | 10 | 12 | 16 | 9 | 13 | 15 | 16 | 14 | 12.50 | |
| Model5 | | | | | | | | | | | | | | 15.08 |
| CD46_3O8E-B | 17 | 19 | 14 | 14 | 15 | 15 | 6 | 8 | 17 | 9 | 14 | 19 | 13.92 | |
| CD46_3O8E-D | 19 | 17 | 16 | 14 | 20 | 16 | 2 | 7 | 19 | 13 | 20 | 20 | 15.25 | |
| CD46_5FO8-C | 20 | 20 | 17 | 14 | 16 | 17 | 12 | 10 | 18 | 19 | 13 | 18 | 16.17 | |
| CD46_3L89-R | 18 | 18 | 15 | 14 | 14 | 10 | 8 | 11 | 20 | 20 | 15 | 17 | 15.00 | |

| | | | | | | | | | | | | | | |
|---|---|---|---|---|---|---|---|---|---|---|---|---|---|---|
| SARS-CoV-2 Envelope Protein (EP) | | | | | | | | | | | | | | |
| Model1 | | | | | | | | | | | | | | 3.90 |
| EP_7K3G-A | — | — | 5 | 4 | 6 | 3 | 7 | 5 | 2 | 2 | 2 | 3 | 3.90 | |
| Model2 | | | | | | | | | | | | | | 6.80 |
| EP_7K3G-A | — | — | 6 | 6 | 7 | 7 | 5 | 7 | 8 | 7 | 8 | 7 | 6.80 | |
| Model3 | | | | | | | | | | | | | | 4.10 |
| EP_7K3G-A | — | — | 4 | 5 | 3 | 4 | 6 | 4 | 5 | 5 | 3 | 2 | 4.10 | |
| Model4 | | | | | | | | | | | | | | 5.60 |
| EP_7K3G-A | — | — | 7 | 7 | 5 | 6 | 4 | 6 | 6 | 6 | 5 | 4 | 5.60 | |
| Model5 | | | | | | | | | | | | | | 7.80 |
| EP_7K3G-A | — | — | 8 | 8 | 8 | 8 | 8 | 8 | 7 | 8 | 7 | 8 | 7.80 | |
| QHD43418 | | | | | | | | | | | | | | **2.10** |
| EP_7K3G-A | — | — | 3 | 3 | 4 | 1 | 3 | 3 | 1 | 1 | 1 | 1 | 2.10 | |
| SARS-CoV-2 Nucleocapsid Protein (NP) | | | | | | | | | | | | | | |
| Model1 | | | | | | | | | | | | | | 21.53 |
| NP_6WZQ-C | — | — | 24 | 16 | 24 | 21 | 34 | 3 | 29 | 27 | 11 | 25 | 21.40 | |
| NP_6YUN-A | — | — | 32 | 16 | 23 | 23 | 35 | 4 | 26 | 25 | 34 | 29 | 24.70 | |
| NP_6YVO-D | — | — | 23 | 16 | 25 | 17 | 15 | 1 | 25 | 28 | 20 | 10 | 18.00 | |
| NP_7CDZ-B | — | — | 26 | 16 | 26 | 16 | 14 | 5 | 30 | 30 | 21 | 18 | 20.20 | |
| NP_7CDZ-D | — | — | 25 | 16 | 27 | 18 | 16 | 2 | 28 | 29 | 30 | 11 | 20.20 | |
| NP_7CE0-C | — | — | 27 | 16 | 22 | 24 | 28 | 12 | 27 | 26 | 35 | 30 | 24.70 | |
| Model2 | | | | | | | | | | | | | | 24.89 |
| NP_6WZQ-C | — | — | 18 | 16 | 17 | 31 | 31 | 7 | 36 | 33 | 12 | 36 | 23.70 | |
| NP_6YUN-A | — | — | 17 | 16 | 28 | 19 | 32 | 6 | 32 | 32 | 16 | 34 | 23.20 | |
| NP_6YVO-D | — | — | — | — | — | — | 33 | 15 | 32 | 34 | 31 | 19 | 27.33 | |
| NP_7CDZ-B | — | — | — | — | — | — | 29 | 8 | 34 | 35 | 26 | 20 | 25.33 | |
| NP_7CDZ-D | — | — | — | — | — | — | 30 | 14 | 34 | 36 | 23 | 20 | 26.17 | |
| NP_7CE0-C | — | — | 19 | 16 | 18 | 32 | 36 | 9 | 31 | 31 | 10 | 34 | 23.60 | |
| Model3 | | | | | | | | | | | | | | 15.43 |
| NP_6WZQ-C | — | — | 22 | 16 | 30 | 28 | 23 | 11 | 17 | 16 | 19 | 33 | 21.50 | |
| NP_6YUN-A | — | — | 21 | 16 | 31 | 30 | 24 | 10 | 18 | 17 | 18 | 32 | 21.70 | |

| | | | | | | | | | | | | | | |
|---|---|---|---|---|---|---|---|---|---|---|---|---|---|---|
| NP_6YVO-D | 6 | 9 | 9 | 15 | 12 | 9 | 3 | 34 | 9 | 8 | 9 | 9 | 11.00 | |
| NP_7CDZ-B | 5 | 8 | 7 | 13 | 10 | 7 | 1 | 27 | 7 | 5 | 7 | 7 | 8.67 | |
| NP_7CDZ-D | 4 | 7 | 8 | 14 | 11 | 8 | 2 | 25 | 8 | 4 | 8 | 8 | 8.92 | |
| NP_7CE0-C | — | — | 20 | 16 | 29 | 22 | 13 | 26 | 16 | 18 | 17 | 31 | 20.80 | |
| | | | | | | Model4 | | | | | | | | 18.41 |
| NP_6WZQ-C | — | — | 29 | 16 | 19 | 25 | 18 | 19 | 21 | 20 | 36 | 26 | 22.90 | |
| NP_6YUN-A | — | — | 30 | 16 | 20 | 26 | 20 | 24 | 19 | 19 | 32 | 28 | 23.40 | |
| NP_6YVO-D | 12 | 14 | 15 | 5 | 7 | 13 | 6 | 23 | 14 | 14 | 23 | 17 | 13.58 | |
| NP_7CDZ-B | 10 | 10 | 13 | 6 | 8 | 11 | 4 | 36 | 10 | 13 | 22 | 12 | 12.92 | |
| NP_7CDZ-D | 11 | 12 | 11 | 8 | 9 | 14 | 5 | 32 | 11 | 11 | 25 | 15 | 13.67 | |
| NP_7CE0-C | — | — | 28 | 16 | 21 | 27 | 17 | 30 | 20 | 21 | 33 | 27 | 24.00 | |
| | | | | | | Model5 | | | | | | | | 18.79 |
| NP_6WZQ-C | — | — | 31 | 16 | 33 | 33 | 27 | 17 | 24 | 23 | 14 | 24 | 24.20 | |
| NP_6YUN-A | — | — | 33 | 16 | 32 | 29 | 26 | 20 | 22 | 22 | 13 | 22 | 23.50 | |
| NP_6YVO-D | 15 | 14 | 14 | 7 | 13 | 15 | 7 | 21 | 15 | 15 | 26 | 16 | 14.83 | |
| NP_7CDZ-B | 13 | 11 | 12 | 9 | 14 | 10 | 9 | 31 | 13 | 12 | 29 | 13 | 14.67 | |
| NP_7CDZ-D | 14 | 12 | 10 | 10 | 15 | 12 | 8 | 33 | 12 | 10 | 28 | 14 | 14.83 | |
| NP_7CE0-C | — | — | 16 | 16 | 16 | 20 | 25 | 29 | 23 | 24 | 15 | 23 | 20.70 | |
| | | | | | | QHD43423 | | | | | | | | **6.36** |
| NP_6WZQ-C | 8 | 3 | 4 | 2 | 2 | 3 | 19 | 13 | 1 | 7 | 3 | 3 | 5.67 | |
| NP_6YUN-A | 7 | 1 | 2 | 1 | 1 | 1 | 21 | 16 | 1 | 6 | 1 | 1 | 4.92 | |
| NP_6YVO-D | 2 | 4 | 1 | 4 | 4 | 4 | 11 | 35 | 1 | 1 | 4 | 5 | 6.33 | |
| NP_7CDZ-B | 3 | 6 | 5 | 11 | 5 | 6 | 12 | 21 | 6 | 3 | 6 | 6 | 7.50 | |
| NP_7CDZ-D | 1 | 5 | 3 | 12 | 6 | 5 | 10 | 18 | 5 | 2 | 5 | 4 | 6.33 | |
| NP_7CE0-C | 9 | 2 | 6 | 3 | 3 | 2 | 22 | 28 | 1 | 9 | 2 | 2 | 7.42 | |
| | | | | | | SARS-CoV-2 Spike Protein (SP) | | | | | | | | |
| | | | | | | Model1 | | | | | | | | 40.45 |
| SP_6XR8-A | 47 | 4 | 64 | 59 | 54 | 25 | 66 | 69 | 1 | 27 | 38 | 52 | 42.17 | |
| SP_6XR8-B | 25 | 24 | 58 | 59 | 68 | 61 | 15 | 19 | 1 | 10 | 33 | 48 | 35.08 | |
| SP_6XR8-C | 22 | 9 | 56 | 59 | 63 | 54 | 15 | 20 | 1 | 9 | 32 | 49 | 32.38 | |
| SP_7DDD-A | 30 | 49 | 66 | 59 | 66 | 67 | 69 | 67 | 1 | 34 | 43 | 57 | 50.63 | |

| | | | | | | | | | | | | | |
|---|---|---|---|---|---|---|---|---|---|---|---|---|---|
| SP_7DDD-B | 31 | 48 | 69 | 59 | 57 | 8 | 46 | 38 | 1 | 60 | 11 | 2 | 35.83 | |
| SP_7DDD-C | 31 | 51 | 66 | 59 | 66 | 67 | 65 | 66 | 1 | 32 | 34 | 54 | 49.29 | |
| SP-6ZWV-A | 71 | 46 | 16 | 24 | 23 | 17 | 41 | 49 | 64 | 46 | 70 | 59 | 43.83 | |
| SP-6ZWV-B | 68 | 39 | 17 | 18 | 24 | 15 | 70 | 59 | 72 | 69 | 72 | 72 | 49.58 | |
| SP-6ZWV-C | 59 | 52 | 18 | 17 | 20 | 20 | 33 | 35 | 65 | 55 | 7 | 63 | 37.00 | |
| SP_7JJI-A | 13 | 62 | 57 | 42 | 60 | 60 | 25 | 39 | 55 | 51 | 24 | 50 | 44.79 | |
| SP_7JJI-B | 37 | 26 | 59 | 59 | 64 | 58 | 12 | 12 | 1 | 5 | 27 | 43 | 33.58 | |
| SP_7JJI-C | 9 | 28 | 59 | 59 | 64 | 58 | 11 | 10 | 1 | 6 | 27 | 43 | 31.25 | |
| | | | | | | Model2 | | | | | | | | 32.16 |
| SP_6XR8-A | 53 | 36 | 31 | 19 | 17 | 27 | 67 | 68 | 1 | 28 | 49 | 9 | 33.75 | |
| SP_6XR8-B | 31 | 5 | 23 | 25 | 19 | 46 | 24 | 23 | 1 | 11 | 42 | 25 | 22.88 | |
| SP_6XR8-C | 31 | 11 | 28 | 29 | 31 | 52 | 23 | 23 | 1 | 12 | 38 | 25 | 25.29 | |
| SP_7DDD-A | 52 | 33 | 41 | 37 | 36 | 43 | 58 | 57 | 1 | 40 | 53 | 37 | 40.67 | |
| SP_7DDD-B | 49 | 61 | 71 | 59 | 58 | 35 | 47 | 38 | 57 | 65 | 65 | 7 | 50.96 | |
| SP_7DDD-C | 49 | 41 | 42 | 36 | 37 | 44 | 51 | 56 | 1 | 36 | 53 | 13 | 38.25 | |
| SP-6ZWV-A | 61 | 45 | 5 | 5 | 6 | 21 | 42 | 44 | 1 | 45 | 3 | 40 | 26.46 | |
| SP-6ZWV-B | 60 | 2 | 4 | 3 | 4 | 19 | 64 | 52 | 67 | 68 | 6 | 67 | 34.67 | |
| SP-6ZWV-C | 56 | 44 | 6 | 4 | 5 | 18 | 32 | 33 | 68 | 59 | 35 | 70 | 35.83 | |
| SP_7JJI-A | 25 | 63 | 25 | 21 | 33 | 47 | 29 | 40 | 1 | 49 | 30 | 36 | 33.25 | |
| SP_7JJI-B | 44 | 16 | 21 | 30 | 26 | 37 | 5 | 7 | 1 | 8 | 53 | 31 | 23.21 | |
| SP_7JJI-C | 22 | 16 | 21 | 30 | 26 | 37 | 2 | 3 | 1 | 7 | 53 | 31 | 20.71 | |
| | | | | | | Model3 | | | | | | | | 33.40 |
| SP_6XR8-A | 53 | 35 | 29 | 23 | 18 | 26 | 59 | 65 | 1 | 31 | 61 | 12 | 34.38 | |
| SP_6XR8-B | 37 | 20 | 26 | 26 | 22 | 31 | 18 | 16 | 1 | 21 | 46 | 29 | 24.38 | |
| SP_6XR8-C | 31 | 11 | 33 | 27 | 28 | 31 | 18 | 16 | 1 | 22 | 45 | 29 | 24.33 | |
| SP_7DDD-A | 46 | 40 | 44 | 47 | 50 | 39 | 60 | 59 | 1 | 42 | 59 | 33 | 43.33 | |
| SP_7DDD-B | 43 | 56 | 72 | 59 | 71 | 29 | 48 | 38 | 61 | 64 | 44 | 62 | 53.88 | |
| SP_7DDD-C | 49 | 43 | 43 | 45 | 50 | 40 | 54 | 58 | 1 | 37 | 59 | 10 | 40.75 | |
| SP-6ZWV-A | 65 | 50 | 10 | 12 | 10 | 12 | 43 | 43 | 1 | 47 | 16 | 56 | 30.42 | |
| SP-6ZWV-B | 64 | 2 | 11 | 10 | 10 | 13 | 53 | 46 | 70 | 71 | 18 | 70 | 36.50 | |
| SP-6ZWV-C | 58 | 60 | 12 | 10 | 10 | 11 | 35 | 33 | 63 | 58 | 10 | 68 | 35.67 | |

| | | | | | | | | | | | | | |
|---|---|---|---|---|---|---|---|---|---|---|---|---|---|
| SP_7JJI-A | 7 | 64 | 24 | 20 | 25 | 30 | 30 | 40 | 54 | 54 | 30 | 34 | 34.29 | |
| SP_7JJI-B | 44 | 8 | 19 | 35 | 32 | 31 | 5 | 8 | 1 | 23 | 46 | 23 | 22.88 | |
| SP_7JJI-C | 14 | 9 | 19 | 34 | 30 | 34 | 3 | 4 | 1 | 24 | 46 | 23 | 20.04 | |
| Model4 | | | | | | | | | | | | | | 36.53 |
| SP_6XR8-A | 27 | 42 | 45 | 44 | 47 | 48 | 68 | 68 | 1 | 33 | 64 | 11 | 41.46 | |
| SP_6XR8-B | 1 | 13 | 37 | 55 | 59 | 70 | 21 | 18 | 1 | 16 | 62 | 16 | 30.75 | |
| SP_6XR8-C | 4 | 13 | 40 | 58 | 61 | 69 | 22 | 20 | 1 | 15 | 62 | 16 | 31.71 | |
| SP_7DDD-A | 21 | 33 | 47 | 50 | 55 | 50 | 57 | 52 | 1 | 41 | 49 | 25 | 40.08 | |
| SP_7DDD-B | 19 | 67 | 70 | 59 | 62 | 36 | 39 | 33 | 53 | 66 | 19 | 4 | 43.88 | |
| SP_7DDD-C | 19 | 38 | 46 | 48 | 56 | 51 | 50 | 55 | 1 | 39 | 49 | 8 | 38.29 | |
| SP-6ZWV-A | 63 | 47 | 8 | 14 | 14 | 10 | 37 | 39 | 1 | 50 | 8 | 38 | 27.42 | |
| SP-6ZWV-B | 62 | 1 | 9 | 15 | 15 | 16 | 56 | 46 | 58 | 72 | 9 | 61 | 34.96 | |
| SP-6ZWV-C | 67 | 32 | 7 | 15 | 15 | 14 | 31 | 32 | 66 | 62 | 14 | 64 | 34.88 | |
| SP_7JJI-A | 10 | 66 | 51 | 46 | 72 | 71 | 27 | 36 | 56 | 52 | 68 | 18 | 47.71 | |
| SP_7JJI-B | 40 | 21 | 49 | 59 | 69 | 65 | 8 | 7 | 1 | 17 | 66 | 21 | 35.21 | |
| SP_7JJI-C | 10 | 21 | 49 | 59 | 69 | 65 | 4 | 3 | 1 | 17 | 66 | 21 | 32.04 | |
| Model5 | | | | | | | | | | | | | | **31.34** |
| SP_6XR8-A | 47 | 36 | 36 | 32 | 20 | 28 | 62 | 65 | 1 | 30 | 49 | 5 | 34.25 | |
| SP_6XR8-B | 15 | 7 | 27 | 33 | 34 | 49 | 17 | 17 | 1 | 14 | 38 | 14 | 22.13 | |
| SP_6XR8-C | 15 | 5 | 32 | 38 | 39 | 62 | 18 | 18 | 1 | 13 | 38 | 15 | 24.50 | |
| SP_7DDD-A | 31 | 31 | 39 | 43 | 43 | 42 | 55 | 54 | 1 | 38 | 58 | 35 | 39.17 | |
| SP_7DDD-B | 37 | 69 | 68 | 59 | 39 | 9 | 44 | 35 | 1 | 63 | 12 | 1 | 36.38 | |
| SP_7DDD-C | 42 | 30 | 38 | 41 | 43 | 41 | 49 | 55 | 1 | 35 | 53 | 6 | 36.13 | |
| SP-6ZWV-A | 69 | 55 | 1 | 9 | 7 | 5 | 38 | 40 | 1 | 48 | 2 | 39 | 26.17 | |
| SP-6ZWV-B | 70 | 68 | 2 | 7 | 7 | 6 | 52 | 45 | 69 | 70 | 14 | 66 | 39.67 | |
| SP-6ZWV-C | 57 | 57 | 3 | 8 | 9 | 7 | 36 | 35 | 62 | 56 | 5 | 65 | 33.29 | |
| SP_7JJI-A | 5 | 65 | 30 | 22 | 35 | 45 | 28 | 37 | 52 | 53 | 29 | 25 | 35.50 | |
| SP_7JJI-B | 41 | 13 | 34 | 39 | 45 | 56 | 5 | 6 | 1 | 19 | 35 | 19 | 26.08 | |
| SP_7JJI-C | 7 | 16 | 34 | 39 | 45 | 56 | 1 | 2 | 1 | 20 | 35 | 19 | 22.88 | |
| QHD43416 | | | | | | | | | | | | | | 34.90 |
| SP_6XR8-A | 22 | 59 | 61 | 54 | 38 | 22 | 63 | 67 | 1 | 25 | 24 | 51 | 40.58 | |

| | | | | | | | | | | | | |
|---|---|---|---|---|---|---|---|---|---|---|---|---|---|
| SP_6XR8-B | 1 | 23 | 48 | 49 | 48 | 55 | 13 | 16 | 1 | 3 | 22 | 47 | 27.17 |
| SP_6XR8-C | 1 | 25 | 52 | 57 | 49 | 53 | 14 | 17 | 1 | 4 | 23 | 45 | 28.42 |
| SP_7DDD-A | 29 | 54 | 62 | 56 | 42 | 24 | 71 | 67 | 1 | 29 | 13 | 58 | 42.17 |
| SP_7DDD-B | 17 | 71 | 65 | 53 | 29 | 1 | 45 | 37 | 1 | 61 | 4 | 3 | 32.25 |
| SP_7DDD-C | 17 | 53 | 63 | 51 | 41 | 23 | 60 | 63 | 1 | 26 | 26 | 53 | 39.71 |
| SP-6ZWV-A | 72 | 58 | 15 | 6 | 1 | 2 | 40 | 48 | 59 | 44 | 69 | 55 | 39.04 |
| SP-6ZWV-B | 66 | 29 | 14 | 2 | 3 | 4 | 72 | 61 | 71 | 67 | 71 | 69 | 44.08 |
| SP-6ZWV-C | 55 | 70 | 13 | 1 | 1 | 3 | 34 | 35 | 60 | 57 | 1 | 60 | 32.50 |
| SP_7JJI-A | 6 | 72 | 53 | 28 | 53 | 64 | 26 | 40 | 51 | 43 | 17 | 46 | 41.58 |
| SP_7JJI-B | 27 | 16 | 54 | 13 | 13 | 72 | 10 | 11 | 1 | 1 | 20 | 41 | 23.21 |
| SP_7JJI-C | 10 | 26 | 55 | 52 | 52 | 63 | 9 | 8 | 1 | 1 | 20 | 41 | 28.13 |

**Table S16.** Validity Assessment of Homology-Based Full-Size 3D Models of SARS-CoV-2 Membrane Protein (MP).

| Model | QMEAN | Model | QMEAN |
|---|---|---|---|
| Model1 | -10.78 | Model4 | -16.07 |
| Model2 | -12.58 | Model5 | -11.80 |
| Mode3 | -12.65 | **QHD43419** | **-5.79** |

**Table S17.** Energetic Characteristics of Protein-Protein Complexes of Human CD46 and SARS-CoV-2 Envelope Protein (EP), Membrane Protein (MP), Nucleocapsid Protein (NP), and Spike Protein (SP).

| Complex | Program | ΔE, kcal/mol | Complex | Program | ΔE, kcal/mol |
|---|---|---|---|---|---|
| CD46 receptor — EP, MP, NP, SP ligands | | | EP, MP, NP, SP receptors — CD46 ligand | | |
| CD46—EP | Cluspro2 | -1218 | EP—CD46 | Cluspro2 | -1217 |
| CD46—EP | GRAMM1 | 20558 | EP—CD46 | GRAMM1 | 20558 |
| CD46—EP | Hex8 | -286.5 | EP—CD46 | Hex8 | -278.8 |
| CD46—EP | SwarmDock | -179.0 | EP—CD46 | SwarmDock | -247.7 |
| CD46—EP | ZDOCK3 | 1815 | EP—CD46 | ZDOCK3 | 1815 |
| CD46—MP | Cluspro2 | -1300 | MP—CD46 | Cluspro2 | -1355 |
| CD46—MP | GRAMM1 | 21735 | MP—CD46 | GRAMM1 | 22061 |
| CD46—MP | Hex8 | -397.2 | MP—CD46 | Hex8 | -412.7 |
| CD46—MP | SwarmDock | -252.3 | MP—CD46 | SwarmDock | -398.7 |
| CD46—MP | ZDOCK3 | 2186 | MP—CD46 | ZDOCK3 | 2186 |
| CD46—NP | Cluspro2 | -1354 | NP—CD46 | Cluspro2 | -1473 |
| CD46—NP | GRAMM1 | 20286 | NP—CD46 | GRAMM1 | 21072 |
| CD46—NP | Hex8 | -468.3 | NP—CD46 | Hex8 | -467.1 |
| CD46—NP | SwarmDock | -193.5 | NP—CD46 | SwarmDock | -178.9 |
| CD46—NP | ZDOCK3 | 1834 | NP—CD46 | ZDOCK3 | 1834 |
| CD46—SP | Cluspro2 | -1329 | SP—CD46 | Cluspro2 | -1329 |
| CD46—SP | GRAMM1 | 68467 | SP—CD46 | GRAMM1 | 65242 |
| CD46—SP | Hex8 | -518.3 | SP—CD46 | Hex8 | -493.6 |
| CD46—SP | SwarmDock | -294.2 | SP—CD46 | SwarmDock | -294.2 |
| CD46—SP | ZDOCK3 | 2219 | SP—CD46 | ZDOCK3 | 2219 |

Note: In Table S17, ΔE is expressed in arbitrary units for the ZDOCK3 program.

The primary energy scoring function for the complexes in different programs is defined as follows:

- Cluspro2 - ELowest: Weighted scoring function of the minimum energy for the given valid complex.
- GRAMM1 - ETotal: Scoring function of the minimum total energy for the given valid complex.
- Hex8 - EInteraction: Scoring function of the minimum energy of protein-protein interaction in the given valid complex.
- SwarmDock - EInteraction: Scoring function of the minimum energy of protein-protein interaction in the most representative cluster of the given valid complex.
- ZDOCK3 - ZDOCKScore: Scoring function of the minimum energy of protein-protein interaction in the given valid complex based on the number of amino acid contacts, expressed in arbitrary units.

**Table S18.** Full Lists of Binding Amino Acids in the Human CD46 and SARS-CoV-2 Envelope Protein (EP) Protein-Protein Complex.

| | **Human CD46** | | | **EP SARS-CoV-2** | |
|---|---|---|---|---|---|
| **Amino Acid** | **Position** | **Occurrence** | **Amino Acid** | **Position** | **Occurrence** |
| **THR** | **188** | **3** | **PHE** | **20** | **6** |
| **TYR** | **189** | **3** | **ILE** | **13** | **5** |
| **SER** | **190** | **3** | **VAL** | **5** | **4** |
| **CYS** | **191** | **3** | **THR** | **9** | **4** |
| **PRO** | **193** | **3** | **LEU** | **12** | **4** |
| **PHE** | **200** | **3** | **SER** | **16** | **4** |
| **LEU** | **202** | **3** | **VAL** | **17** | **4** |
| **GLU** | **205** | **3** | **LEU** | **19** | **4** |
| **SER** | **206** | **3** | **PHE** | **23** | **4** |
| **THR** | **207** | **3** | **LEU** | **27** | **4** |
| **TYR** | **247** | **3** | **VAL** | **29** | **4** |
| PRO | 3 | 2 | **LEU** | **34** | **4** |
| PHE | 11 | 2 | **ARG** | **38** | **4** |
| TRP | 14 | 2 | **ASN** | **45** | **4** |
| PHE | 16 | 2 | **ASN** | **66** | **4** |
| PRO | 17 | 2 | MET | 1 | 3 |
| GLY | 18 | 2 | SER | 3 | 3 |
| LEU | 20 | 2 | VAL | 14 | 3 |
| LEU | 21 | 2 | LEU | 18 | 3 |
| MET | 24 | 2 | VAL | 25 | 3 |
| LEU | 27 | 2 | LEU | 28 | 3 |
| TYR | 29 | 2 | THR | 30 | 3 |
| PHE | 31 | 2 | ALA | 32 | 3 |
| ASN | 83 | 2 | THR | 35 | 3 |
| HIS | 84 | 2 | LEU | 39 | 3 |
| THR | 85 | 2 | TYR | 42 | 3 |
| PRO | 88 | 2 | ASN | 48 | 3 |
| VAL | 89 | 2 | VAL | 52 | 3 |
| SER | 90 | 2 | VAL | 70 | 3 |
| PRO | 166 | 2 | LEU | 73 | 3 |
| ILE | 168 | 2 | VAL | 75 | 3 |
| THR | 174 | 2 | PHE | 4 | 2 |
| PHE | 175 | 2 | LEU | 21 | 2 |
| LEU | 184 | 2 | ALA | 22 | 2 |
| ALA | 186 | 2 | VAL | 24 | 2 |
| VAL | 187 | 2 | ILE | 33 | 2 |
| ALA | 194 | 2 | ALA | 36 | 2 |
| PRO | 197 | 2 | CYS | 40 | 2 |
| PRO | 199 | 2 | ALA | 41 | 2 |
| TYR | 209 | 2 | CYS | 43 | 2 |
| TRP | 216 | 2 | CYS | 44 | 2 |
| ALA | 219 | 2 | ILE | 46 | 2 |
| ALA | 220 | 2 | PRO | 54 | 2 |
| GLU | 222 | 2 | TYR | 59 | 2 |
| ILE | 239 | 2 | ARG | 61 | 2 |

| | | |
|---|---|---|
| PHE | 242 | 2 |
| GLY | 243 | 2 |
| LYS | 244 | 2 |
| LYS | 245 | 2 |
| TYR | 248 | 2 |
| SER | 341 | 2 |
| VAL | 351 | 2 |
| ALA | 353 | 2 |
| LYS | 169 | 1 |
| ASP | 192 | 1 |
| ILE | 203 | 1 |
| PRO | 221 | 1 |
| LYS | 237 | 1 |
| GLN | 238 | 1 |
| SER | 240 | 1 |
| GLY | 241 | 1 |
| LYS | 249 | 1 |
| THR | 251 | 1 |
| MET | 253 | 1 |
| GLU | 255 | 1 |
| TYR | 261 | 1 |
| LEU | 262 | 1 |
| ASP | 263 | 1 |
| GLY | 264 | 1 |
| SER | 265 | 1 |
| ASP | 266 | 1 |
| THR | 267 | 1 |
| VAL | 269 | 1 |
| ASP | 271 | 1 |
| ASP | 277 | 1 |
| PRO | 278 | 1 |
| PRO | 279 | 1 |
| PRO | 281 | 1 |
| THR | 292 | 1 |
| LYS | 293 | 1 |
| PRO | 295 | 1 |
| LEU | 297 | 1 |
| HIS | 299 | 1 |
| SER | 300 | 1 |
| THR | 307 | 1 |
| SER | 309 | 1 |
| PRO | 310 | 1 |
| ALA | 314 | 1 |
| GLY | 316 | 1 |
| PRO | 317 | 1 |
| PRO | 323 | 1 |
| SER | 326 | 1 |
| ASN | 327 | 1 |
| GLY | 337 | 1 |
| ILE | 338 | 1 |

| | | |
|---|---|---|
| VAL | 62 | 2 |
| TYR | 2 | 1 |
| SER | 6 | 1 |
| GLU | 8 | 1 |
| THR | 11 | 1 |
| ASN | 15 | 1 |
| PHE | 26 | 1 |
| LEU | 31 | 1 |
| LEU | 37 | 1 |
| VAL | 47 | 1 |
| VAL | 49 | 1 |
| SER | 55 | 1 |
| PHE | 56 | 1 |
| SER | 67 | 1 |
| PRO | 71 | 1 |
| ASP | 72 | 1 |
| LEU | 74 | 1 |

| | | |
|---|---|---|
| LEU | 339 | 1 |
| LEU | 342 | 1 |
| TRP | 345 | 1 |
| ILE | 352 | 1 |
| ILE | 354 | 1 |
| VAL | 355 | 1 |
| VAL | 358 | 1 |
| ALA | 359 | 1 |
| VAL | 360 | 1 |
| CYS | 362 | 1 |
| THR | 383 | 1 |
| ARG | 385 | 1 |
| VAL | 387 | 1 |

Note: Binding amino acids with the highest occurrence are highlighted in bold.

**Table S19.** Full Lists of Binding Amino Acids in the Human CD46 and SARS-CoV-2 Membrane Protein (MP) Protein-Protein Complex.

| | **Human CD46** | | | **MP SARS-CoV-2** | |
|---|---|---|---|---|---|
| **Amino Acid** | **Position** | **Occurrence** | **Amino Acid** | **Position** | **Occurrence** |
| **PHE** | **11** | **4** | **VAL** | **60** | **7** |
| **PRO** | **12** | **4** | **PRO** | **59** | **6** |
| **TRP** | **14** | **4** | **ALA** | **63** | **6** |
| **PHE** | **16** | **4** | **VAL** | **66** | **6** |
| **PRO** | **17** | **4** | **VAL** | **70** | **6** |
| **LEU** | **20** | **4** | **ASP** | **3** | **5** |
| **MET** | **24** | **4** | **LEU** | **56** | **5** |
| **VAL** | **25** | **4** | **LEU** | **67** | **5** |
| **LEU** | **26** | **4** | **PHE** | **96** | **5** |
| **LEU** | **27** | **4** | **MET** | **1** | **4** |
| **TYR** | **29** | **4** | **LEU** | **13** | **4** |
| **PHE** | **31** | **4** | **TRP** | **20** | **4** |
| **GLU** | **36** | **4** | **LEU** | **57** | **4** |
| **ASN** | **83** | **4** | **LEU** | **62** | **4** |
| **THR** | **85** | **4** | **TYR** | **71** | **4** |
| **ILE** | **239** | **4** | **ILE** | **82** | **4** |
| **TYR** | **247** | **4** | **LEU** | **90** | **4** |
| **THR** | **251** | **4** | **TRP** | **92** | **4** |
| **VAL** | **269** | **4** | LEU | 17 | 3 |
| SER | 30 | 3 | ILE | 24 | 3 |
| HIS | 84 | 3 | GLN | 36 | 3 |
| LYS | 237 | 3 | TYR | 39 | 3 |
| SER | 240 | 3 | PHE | 45 | 3 |
| GLY | 241 | 3 | ILE | 49 | 3 |
| PHE | 242 | 3 | TRP | 55 | 3 |
| LYS | 249 | 3 | CYS | 64 | 3 |
| MET | 253 | 3 | ALA | 69 | 3 |
| SER | 265 | 3 | ARG | 72 | 3 |
| ASP | 266 | 3 | MET | 91 | 3 |

| | | | | | |
|---|---|---|---|---|---|
| THR | 267 | 3 | SER | 94 | 3 |
| LEU | 342 | 3 | TYR | 95 | 3 |
| VAL | 351 | 3 | MET | 109 | 3 |
| VAL | 355 | 3 | PHE | 112 | 3 |
| PRO | 3 | 2 | LEU | 134 | 3 |
| SER | 13 | 2 | GLU | 135 | 3 |
| ARG | 15 | 2 | ALA | 2 | 2 |
| LEU | 21 | 2 | SER | 4 | 2 |
| LEU | 28 | 2 | THR | 7 | 2 |
| SER | 32 | 2 | ILE | 8 | 2 |
| ASP | 33 | 2 | VAL | 10 | 2 |
| ALA | 34 | 2 | LEU | 16 | 2 |
| LEU | 184 | 2 | TRP | 31 | 2 |
| ALA | 186 | 2 | ILE | 32 | 2 |
| THR | 188 | 2 | PHE | 37 | 2 |
| SER | 190 | 2 | ALA | 40 | 2 |
| LEU | 202 | 2 | ASN | 43 | 2 |
| GLU | 205 | 2 | ARG | 44 | 2 |
| SER | 206 | 2 | ILE | 52 | 2 |
| THR | 207 | 2 | PHE | 53 | 2 |
| GLU | 255 | 2 | TRP | 58 | 2 |
| ASP | 257 | 2 | PHE | 65 | 2 |
| LYS | 258 | 2 | TRP | 75 | 2 |
| TYR | 261 | 2 | CYS | 86 | 2 |
| GLY | 264 | 2 | LEU | 93 | 2 |
| ALA | 314 | 2 | ILE | 97 | 2 |
| PRO | 323 | 2 | PHE | 100 | 2 |
| LEU | 339 | 2 | PHE | 103 | 2 |
| ASP | 340 | 2 | ARG | 107 | 2 |
| SER | 341 | 2 | GLU | 115 | 2 |
| VAL | 344 | 2 | ASN | 117 | 2 |
| TRP | 345 | 2 | HIS | 125 | 2 |
| ILE | 347 | 2 | ASP | 163 | 2 |
| ALA | 348 | 2 | PRO | 165 | 2 |
| ALA | 353 | 2 | GLU | 167 | 2 |
| VAL | 360 | 2 | ARG | 174 | 2 |
| HIS | 384 | 2 | TYR | 178 | 2 |
| PRO | 4 | 1 | GLU | 12 | 1 |
| ARG | 6 | 1 | LEU | 27 | 1 |
| CYS | 9 | 1 | CYS | 33 | 1 |
| PRO | 52 | 1 | LEU | 35 | 1 |
| ASP | 104 | 1 | ILE | 48 | 1 |
| ASN | 107 | 1 | LYS | 50 | 1 |
| ASN | 128 | 1 | LEU | 54 | 1 |
| GLU | 129 | 1 | THR | 61 | 1 |
| TYR | 132 | 1 | ILE | 73 | 1 |
| THR | 163 | 1 | ASN | 74 | 1 |
| LYS | 172 | 1 | GLY | 78 | 1 |
| PHE | 175 | 1 | LEU | 87 | 1 |
| TYR | 183 | 1 | ARG | 101 | 1 |

| | | | | | |
|---|---|---|---|---|---|
| ASP | 185 | 1 | ARG | 105 | 1 |
| TYR | 189 | 1 | PRO | 123 | 1 |
| CYS | 191 | 1 | GLY | 126 | 1 |
| ASP | 192 | 1 | ILE | 128 | 1 |
| PRO | 193 | 1 | GLY | 141 | 1 |
| ALA | 194 | 1 | VAL | 143 | 1 |
| PRO | 199 | 1 | ARG | 150 | 1 |
| ILE | 203 | 1 | ILE | 151 | 1 |
| VAL | 215 | 1 | ALA | 152 | 1 |
| ALA | 219 | 1 | GLY | 153 | 1 |
| ALA | 220 | 1 | ARG | 158 | 1 |
| GLU | 222 | 1 | LYS | 162 | 1 |
| LYS | 224 | 1 | SER | 173 | 1 |
| GLY | 243 | 1 | LEU | 176 | 1 |
| LYS | 244 | 1 | GLN | 185 | 1 |
| TYR | 248 | 1 | PHE | 193 | 1 |
| ALA | 250 | 1 | ILE | 201 | 1 |
| LEU | 262 | 1 | GLY | 202 | 1 |
| ASP | 263 | 1 | ASN | 203 | 1 |
| SER | 272 | 1 | TYR | 204 | 1 |
| PRO | 278 | 1 | LYS | 205 | 1 |
| HIS | 299 | 1 | GLN | 222 | 1 |
| SER | 315 | 1 | | | |
| GLY | 316 | 1 | | | |
| PRO | 317 | 1 | | | |
| THR | 320 | 1 | | | |
| LYS | 322 | 1 | | | |
| SER | 326 | 1 | | | |
| ASP | 343 | 1 | | | |
| GLY | 357 | 1 | | | |

Note: Binding amino acids with the highest occurrence are highlighted in bold.

**Table S20.** Full Lists of Binding Amino Acids in the Human CD46 and SARS-CoV-2 Nucleocapsid Protein (NP) Protein-Protein Complex.

| | **Human CD46** | | | **NP SARS-CoV-2** | |
|---|---|---|---|---|---|
| **Amino Acid** | **Position** | **Occurrence** | **Amino Acid** | **Position** | **Occurrence** |
| **ASP** | **343** | **5** | **PHE** | **17** | **4** |
| **VAL** | **344** | **5** | **SER** | **21** | **4** |
| **TRP** | **345** | **5** | **SER** | **79** | **4** |
| **TYR** | **247** | **4** | **HIS** | **145** | **4** |
| **SER** | **341** | **4** | **GLY** | **212** | **4** |
| **LEU** | **342** | **4** | **ASP** | **216** | **4** |
| **VAL** | **346** | **4** | **THR** | **334** | **4** |
| **VAL** | **351** | **4** | **GLY** | **335** | **4** |
| **TYR** | **132** | **3** | **ALA** | **336** | **4** |
| **ALA** | **194** | **3** | **ASP** | **81** | **3** |
| **PRO** | **199** | **3** | **ASP** | **82** | **3** |
| **GLU** | **205** | **3** | **ASN** | **140** | **3** |

| | | | | | |
|---|---|---|---|---|---|
| **TRP** | **216** | **3** | **THR** | **141** | **3** |
| **ARG** | **218** | **3** | **ALA** | **211** | **3** |
| **PHE** | **242** | **3** | **ASN** | **213** | **3** |
| **LYS** | **245** | **3** | **LEU** | **219** | **3** |
| **ILE** | **338** | **3** | **LEU** | **223** | **3** |
| **LEU** | **339** | **3** | **ARG** | **259** | **3** |
| **ASP** | **340** | **3** | **ARG** | **277** | **3** |
| **ILE** | **347** | **3** | **PRO** | **279** | **3** |
| **ALA** | **353** | **3** | **GLN** | **281** | **3** |
| **ILE** | **354** | **3** | **THR** | **282** | **3** |
| **VAL** | **355** | **3** | **GLN** | **283** | **3** |
| **ALA** | **359** | **3** | **GLY** | **284** | **3** |
| **VAL** | **360** | **3** | **ASN** | **285** | **3** |
| **TYR** | **366** | **3** | **PHE** | **286** | **3** |
| **TYR** | **368** | **3** | **ILE** | **304** | **3** |
| **TYR** | **378** | **3** | **PRO** | **309** | **3** |
| **THR** | **380** | **3** | **PHE** | **314** | **3** |
| **THR** | **383** | **3** | **TYR** | **333** | **3** |
| **HIS** | **384** | **3** | **ILE** | **337** | **3** |
| MET | 1 | 2 | GLN | 7 | 2 |
| GLU | 2 | 2 | GLN | 9 | 2 |
| PRO | 3 | 2 | ARG | 14 | 2 |
| GLY | 5 | 2 | ILE | 15 | 2 |
| ARG | 6 | 2 | THR | 16 | 2 |
| ARG | 7 | 2 | ASN | 77 | 2 |
| GLU | 8 | 2 | SER | 78 | 2 |
| CYS | 9 | 2 | ARG | 92 | 2 |
| PHE | 11 | 2 | ILE | 94 | 2 |
| TRP | 14 | 2 | ARG | 95 | 2 |
| ARG | 15 | 2 | LEU | 104 | 2 |
| PHE | 16 | 2 | ARG | 107 | 2 |
| PRO | 17 | 2 | LEU | 139 | 2 |
| LEU | 19 | 2 | VAL | 158 | 2 |
| LEU | 20 | 2 | ARG | 177 | 2 |
| LEU | 21 | 2 | GLN | 181 | 2 |
| ALA | 22 | 2 | ARG | 195 | 2 |
| ALA | 23 | 2 | THR | 198 | 2 |
| MET | 24 | 2 | SER | 201 | 2 |
| VAL | 25 | 2 | PRO | 207 | 2 |
| LEU | 27 | 2 | ALA | 208 | 2 |
| TYR | 29 | 2 | ARG | 226 | 2 |
| LEU | 133 | 2 | LEU | 227 | 2 |
| GLY | 135 | 2 | LEU | 230 | 2 |
| GLU | 137 | 2 | GLU | 231 | 2 |
| THR | 163 | 2 | GLN | 239 | 2 |
| SER | 190 | 2 | GLN | 241 | 2 |
| ASP | 192 | 2 | GLN | 244 | 2 |
| PRO | 193 | 2 | THR | 245 | 2 |
| GLY | 196 | 2 | VAL | 246 | 2 |
| PHE | 200 | 2 | GLN | 260 | 2 |

| | | | | | |
|---|---|---|---|---|---|
| ILE | 203 | 2 | ARG | 262 | 2 |
| TYR | 209 | 2 | PHE | 274 | 2 |
| ASN | 213 | 2 | GLU | 280 | 2 |
| VAL | 215 | 2 | THR | 296 | 2 |
| SER | 217 | 2 | TRP | 301 | 2 |
| ALA | 219 | 2 | ALA | 305 | 2 |
| GLY | 243 | 2 | PHE | 307 | 2 |
| TYR | 248 | 2 | ALA | 308 | 2 |
| LYS | 249 | 2 | MET | 317 | 2 |
| THR | 251 | 2 | SER | 318 | 2 |
| ALA | 314 | 2 | ARG | 319 | 2 |
| PRO | 317 | 2 | GLU | 323 | 2 |
| SER | 326 | 2 | SER | 327 | 2 |
| CYS | 362 | 2 | TRP | 330 | 2 |
| PRO | 365 | 2 | LEU | 331 | 2 |
| ARG | 367 | 2 | LEU | 339 | 2 |
| GLN | 370 | 2 | LEU | 353 | 2 |
| ARG | 372 | 2 | ILE | 357 | 2 |
| LYS | 374 | 2 | THR | 393 | 2 |
| LEU | 379 | 2 | LEU | 394 | 2 |
| ASP | 381 | 2 | LEU | 395 | 2 |
| GLU | 382 | 2 | LEU | 400 | 2 |
| ARG | 385 | 2 | PHE | 403 | 2 |
| GLU | 386 | 2 | SER | 404 | 2 |
| VAL | 387 | 2 | LEU | 407 | 2 |
| PRO | 4 | 1 | MET | 411 | 2 |
| PRO | 10 | 1 | ASN | 11 | 1 |
| PRO | 12 | 1 | SER | 23 | 1 |
| GLY | 18 | 1 | ASN | 29 | 1 |
| LEU | 26 | 1 | GLY | 30 | 1 |
| TYR | 101 | 1 | ARG | 32 | 1 |
| GLN | 109 | 1 | SER | 33 | 1 |
| ALA | 110 | 1 | GLY | 34 | 1 |
| VAL | 111 | 1 | ARG | 36 | 1 |
| PRO | 112 | 1 | SER | 37 | 1 |
| TYR | 117 | 1 | LYS | 38 | 1 |
| HIS | 124 | 1 | ARG | 40 | 1 |
| ILE | 126 | 1 | ARG | 41 | 1 |
| CYS | 127 | 1 | LYS | 61 | 1 |
| ILE | 134 | 1 | ASP | 63 | 1 |
| LEU | 161 | 1 | LYS | 65 | 1 |
| PRO | 166 | 1 | THR | 76 | 1 |
| ILE | 168 | 1 | PRO | 80 | 1 |
| THR | 174 | 1 | ILE | 84 | 1 |
| PHE | 175 | 1 | ARG | 93 | 1 |
| THR | 188 | 1 | LYS | 100 | 1 |
| CYS | 191 | 1 | GLY | 114 | 1 |
| PRO | 197 | 1 | ALA | 119 | 1 |
| SER | 201 | 1 | GLY | 120 | 1 |
| LEU | 202 | 1 | TYR | 123 | 1 |

| | | |
|---|---|---|
| GLY | 204 | 1 |
| GLY | 211 | 1 |
| SER | 214 | 1 |
| ALA | 220 | 1 |
| GLU | 222 | 1 |
| GLN | 238 | 1 |
| SER | 240 | 1 |
| GLY | 241 | 1 |
| PHE | 246 | 1 |
| ALA | 250 | 1 |
| MET | 253 | 1 |
| GLU | 255 | 1 |
| TYR | 261 | 1 |
| ASP | 266 | 1 |
| THR | 267 | 1 |
| ASP | 271 | 1 |
| SER | 272 | 1 |
| ASN | 273 | 1 |
| THR | 275 | 1 |
| TRP | 276 | 1 |
| ASP | 277 | 1 |
| PRO | 279 | 1 |
| PRO | 310 | 1 |
| SER | 313 | 1 |
| SER | 315 | 1 |
| GLY | 316 | 1 |
| TYR | 321 | 1 |
| LYS | 322 | 1 |
| ASN | 327 | 1 |
| TYR | 328 | 1 |
| TYR | 331 | 1 |
| GLY | 357 | 1 |
| VAL | 358 | 1 |

| | | |
|---|---|---|
| GLY | 124 | 1 |
| ALA | 125 | 1 |
| ASN | 126 | 1 |
| LYS | 127 | 1 |
| ASP | 128 | 1 |
| ILE | 130 | 1 |
| ILE | 131 | 1 |
| GLU | 136 | 1 |
| GLY | 137 | 1 |
| ALA | 138 | 1 |
| PRO | 142 | 1 |
| LYS | 143 | 1 |
| ASP | 144 | 1 |
| ALA | 173 | 1 |
| ARG | 191 | 1 |
| ASN | 192 | 1 |
| SER | 193 | 1 |
| SER | 194 | 1 |
| ASN | 196 | 1 |
| SER | 197 | 1 |
| SER | 206 | 1 |
| ARG | 209 | 1 |
| MET | 210 | 1 |
| ALA | 217 | 1 |
| ALA | 220 | 1 |
| PRO | 258 | 1 |
| LYS | 261 | 1 |
| THR | 263 | 1 |
| ALA | 264 | 1 |
| VAL | 270 | 1 |
| ALA | 273 | 1 |
| GLY | 278 | 1 |
| LEU | 291 | 1 |
| GLN | 306 | 1 |
| LYS | 338 | 1 |
| ASP | 341 | 1 |
| LYS | 342 | 1 |
| PRO | 344 | 1 |
| ASN | 345 | 1 |
| LYS | 347 | 1 |
| ASN | 354 | 1 |
| ASP | 358 | 1 |
| LYS | 361 | 1 |
| LYS | 388 | 1 |
| GLN | 390 | 1 |
| VAL | 392 | 1 |
| GLN | 409 | 1 |
| SER | 410 | 1 |
| ASP | 415 | 1 |
| SER | 416 | 1 |

| | | | | | |
|---|---|---|---|---|---|
| | | | GLN | 418 | 1 |
| | | | ALA | 419 | 1 |

Note: Binding amino acids with the highest occurrence are highlighted in bold.

**Table S21.** Full Lists of Binding Amino Acids in the Human CD46 and SARS-CoV-2 Spike Protein (SP) Protein-Protein Complex.

| **Human CD46** | | | **SP SARS-CoV-2** | | |
|---|---|---|---|---|---|
| **Amino Acid** | **Position** | **Occurrence** | **Amino Acid** | **Position** | **Occurrence** |
| **ARG** | **218** | **5** | **VAL** | **3** | **5** |
| **GLN** | **122** | **4** | **LEU** | **5** | **5** |
| **GLU** | **177** | **4** | **MET** | **1** | **4** |
| **ALA** | **219** | **4** | **PHE** | **2** | **4** |
| **LYS** | **249** | **4** | **PHE** | **4** | **3** |
| **TYR** | **131** | **3** | **VAL** | **6** | **3** |
| **VAL** | **178** | **3** | **ASP** | **198** | **3** |
| **PRO** | **199** | **3** | **GLY** | **199** | **3** |
| **GLU** | **205** | **3** | **GLN** | **271** | **3** |
| **VAL** | **215** | **3** | **SER** | **375** | **3** |
| **TRP** | **216** | **3** | **ASP** | **405** | **3** |
| **SER** | **217** | **3** | **ARG** | **408** | **3** |
| **SER** | **240** | **3** | **GLY** | **502** | **3** |
| **PHE** | **242** | **3** | **VAL** | **503** | **3** |
| **LYS** | **244** | **3** | **LEU** | **1145** | **3** |
| **TYR** | **248** | **3** | **ASP** | **1146** | **3** |
| **LYS** | **293** | **3** | ASN | 61 | 2 |
| **PRO** | **295** | **3** | PRO | 85 | 2 |
| **LEU** | **342** | **3** | ASN | 87 | 2 |
| **TRP** | **345** | **3** | LYS | 113 | 2 |
| **ILE** | **347** | **3** | THR | 114 | 2 |
| **VAL** | **351** | **3** | GLN | 115 | 2 |
| **ALA** | **353** | **3** | GLU | 132 | 2 |
| **VAL** | **355** | **3** | ASN | 165 | 2 |
| **GLY** | **357** | **3** | THR | 167 | 2 |
| **ALA** | **359** | **3** | TYR | 200 | 2 |
| **VAL** | **360** | **3** | GLN | 321 | 2 |
| **PHE** | **389** | **3** | GLY | 404 | 2 |
| THR | 40 | 2 | THR | 415 | 2 |
| ILE | 71 | 2 | GLY | 416 | 2 |
| PRO | 72 | 2 | LYS | 417 | 2 |
| PRO | 73 | 2 | ASP | 420 | 2 |
| LEU | 74 | 2 | LEU | 455 | 2 |
| ALA | 75 | 2 | PHE | 456 | 2 |
| PRO | 88 | 2 | TYR | 473 | 2 |
| SER | 90 | 2 | GLN | 474 | 2 |
| ASP | 92 | 2 | ALA | 475 | 2 |
| TYR | 95 | 2 | GLY | 485 | 2 |
| LEU | 106 | 2 | PHE | 486 | 2 |
| TYR | 117 | 2 | ASN | 487 | 2 |
| HIS | 124 | 2 | TYR | 489 | 2 |
| ILE | 126 | 2 | GLN | 493 | 2 |

| | | | | | |
|---|---|---|---|---|---|
| CYS | 127 | 2 | THR | 500 | 2 |
| GLU | 129 | 2 | ASN | 501 | 2 |
| LEU | 133 | 2 | GLY | 504 | 2 |
| GLU | 136 | 2 | ARG | 634 | 2 |
| GLU | 137 | 2 | TYR | 674 | 2 |
| ILE | 138 | 2 | GLU | 725 | 2 |
| THR | 163 | 2 | GLN | 762 | 2 |
| PRO | 166 | 2 | ARG | 765 | 2 |
| ILE | 168 | 2 | PHE | 970 | 2 |
| LYS | 169 | 2 | GLY | 971 | 2 |
| THR | 174 | 2 | ILE | 973 | 2 |
| PHE | 175 | 2 | GLU | 988 | 2 |
| GLU | 179 | 2 | GLN | 992 | 2 |
| TYR | 183 | 2 | ARG | 995 | 2 |
| ASP | 185 | 2 | GLN | 1002 | 2 |
| THR | 188 | 2 | GLU | 1017 | 2 |
| TYR | 189 | 2 | ALA | 1020 | 2 |
| SER | 190 | 2 | LEU | 1024 | 2 |
| LEU | 202 | 2 | ASP | 1041 | 2 |
| GLY | 204 | 2 | LYS | 1149 | 2 |
| SER | 206 | 2 | GLU | 1150 | 2 |
| TYR | 209 | 2 | ASP | 1153 | 2 |
| ASP | 212 | 2 | PHE | 1156 | 2 |
| ASN | 213 | 2 | SER | 1161 | 2 |
| ALA | 220 | 2 | ASN | 1178 | 2 |
| GLU | 222 | 2 | ILE | 1183 | 2 |
| GLN | 238 | 2 | LEU | 1186 | 2 |
| ILE | 239 | 2 | TRP | 1214 | 2 |
| TYR | 247 | 2 | ILE | 1216 | 2 |
| MET | 253 | 2 | TRP | 1217 | 2 |
| ASP | 266 | 2 | LEU | 1218 | 2 |
| THR | 267 | 2 | PHE | 1220 | 2 |
| SER | 290 | 2 | ILE | 1225 | 2 |
| SER | 291 | 2 | MET | 1233 | 2 |
| THR | 292 | 2 | LEU | 1234 | 2 |
| PRO | 294 | 2 | CYS | 1236 | 2 |
| SER | 305 | 2 | CYS | 1250 | 2 |
| THR | 306 | 2 | VAL | 1268 | 2 |
| THR | 307 | 2 | LYS | 1269 | 2 |
| SER | 309 | 2 | HIS | 1271 | 2 |
| SER | 313 | 2 | LEU | 7 | 1 |
| SER | 315 | 2 | LEU | 8 | 1 |
| TYR | 321 | 2 | PRO | 9 | 1 |
| ASN | 327 | 2 | LEU | 10 | 1 |
| TYR | 328 | 2 | SER | 12 | 1 |
| PRO | 329 | 2 | GLN | 14 | 1 |
| TYR | 331 | 2 | ARG | 21 | 1 |
| LYS | 333 | 2 | GLN | 23 | 1 |
| VAL | 344 | 2 | LEU | 24 | 1 |
| VAL | 358 | 2 | PRO | 26 | 1 |

| | | | | | |
|---|---|---|---|---|---|
| CYS | 362 | 2 | TYR | 28 | 1 |
| ARG | 367 | 2 | ASN | 30 | 1 |
| LYS | 375 | 2 | PHE | 59 | 1 |
| THR | 383 | 2 | ASP | 80 | 1 |
| LYS | 388 | 2 | ASN | 81 | 1 |
| SER | 391 | 2 | PRO | 82 | 1 |
| LEU | 392 | 2 | VAL | 83 | 1 |
| ARG | 6 | 1 | LEU | 84 | 1 |
| ARG | 7 | 1 | PHE | 86 | 1 |
| GLU | 8 | 1 | ASP | 88 | 1 |
| CYS | 9 | 1 | THR | 108 | 1 |
| PHE | 11 | 1 | ASN | 196 | 1 |
| TRP | 14 | 1 | ILE | 197 | 1 |
| LEU | 20 | 1 | ASN | 234 | 1 |
| MET | 24 | 1 | VAL | 267 | 1 |
| VAL | 25 | 1 | GLY | 268 | 1 |
| LEU | 28 | 1 | TYR | 269 | 1 |
| TYR | 29 | 1 | PRO | 272 | 1 |
| SER | 30 | 1 | GLU | 309 | 1 |
| GLU | 36 | 1 | LYS | 310 | 1 |
| GLU | 37 | 1 | GLY | 311 | 1 |
| PRO | 38 | 1 | ILE | 312 | 1 |
| LYS | 66 | 1 | ASN | 317 | 1 |
| THR | 85 | 1 | ARG | 319 | 1 |
| TRP | 86 | 1 | PRO | 322 | 1 |
| LEU | 87 | 1 | THR | 323 | 1 |
| THR | 98 | 1 | GLU | 324 | 1 |
| ASN | 107 | 1 | LEU | 335 | 1 |
| VAL | 111 | 1 | CYS | 336 | 1 |
| ALA | 113 | 1 | PRO | 337 | 1 |
| MET | 123 | 1 | GLY | 339 | 1 |
| PHE | 125 | 1 | GLU | 340 | 1 |
| ASN | 128 | 1 | ASN | 343 | 1 |
| TYR | 132 | 1 | ASP | 364 | 1 |
| LEU | 139 | 1 | VAL | 367 | 1 |
| TYR | 140 | 1 | TYR | 369 | 1 |
| GLU | 142 | 1 | SER | 373 | 1 |
| SER | 151 | 1 | PHE | 374 | 1 |
| CYS | 157 | 1 | THR | 376 | 1 |
| GLU | 158 | 1 | PHE | 377 | 1 |
| LYS | 159 | 1 | GLY | 381 | 1 |
| LEU | 161 | 1 | VAL | 382 | 1 |
| LYS | 167 | 1 | SER | 383 | 1 |
| ASN | 170 | 1 | PRO | 384 | 1 |
| VAL | 180 | 1 | THR | 385 | 1 |
| GLU | 182 | 1 | VAL | 407 | 1 |
| LEU | 184 | 1 | ASN | 437 | 1 |
| ASP | 192 | 1 | GLN | 506 | 1 |
| ALA | 194 | 1 | PRO | 527 | 1 |
| ASP | 198 | 1 | LYS | 529 | 1 |

| | | | | | |
|---|---|---|---|---|---|
| PHE | 200 | 1 | ASN | 556 | 1 |
| ILE | 203 | 1 | LYS | 557 | 1 |
| SER | 214 | 1 | ILE | 569 | 1 |
| PRO | 221 | 1 | PHE | 592 | 1 |
| CYS | 223 | 1 | THR | 604 | 1 |
| VAL | 225 | 1 | SER | 605 | 1 |
| LYS | 227 | 1 | ASN | 606 | 1 |
| CYS | 228 | 1 | ASP | 614 | 1 |
| ARG | 229 | 1 | VAL | 615 | 1 |
| PHE | 230 | 1 | ASN | 616 | 1 |
| VAL | 232 | 1 | THR | 618 | 1 |
| LYS | 237 | 1 | HIS | 625 | 1 |
| GLY | 243 | 1 | ALA | 626 | 1 |
| LYS | 245 | 1 | ASP | 627 | 1 |
| THR | 251 | 1 | GLN | 628 | 1 |
| ASP | 257 | 1 | THR | 632 | 1 |
| LYS | 258 | 1 | VAL | 635 | 1 |
| LEU | 262 | 1 | SER | 637 | 1 |
| ASP | 263 | 1 | THR | 638 | 1 |
| SER | 265 | 1 | GLY | 639 | 1 |
| VAL | 269 | 1 | SER | 640 | 1 |
| ASP | 271 | 1 | PHE | 643 | 1 |
| SER | 272 | 1 | GLN | 644 | 1 |
| ASN | 273 | 1 | ARG | 646 | 1 |
| ASP | 277 | 1 | HIS | 655 | 1 |
| PRO | 278 | 1 | VAL | 656 | 1 |
| PRO | 279 | 1 | GLU | 661 | 1 |
| LYS | 282 | 1 | CYS | 662 | 1 |
| LEU | 297 | 1 | ASP | 663 | 1 |
| HIS | 299 | 1 | PRO | 665 | 1 |
| ARG | 318 | 1 | GLY | 667 | 1 |
| LEU | 339 | 1 | ALA | 668 | 1 |
| SER | 341 | 1 | GLY | 669 | 1 |
| ASP | 343 | 1 | SER | 673 | 1 |
| ALA | 348 | 1 | GLN | 675 | 1 |
| VAL | 349 | 1 | GLN | 677 | 1 |
| ILE | 350 | 1 | THR | 678 | 1 |
| ILE | 352 | 1 | ILE | 693 | 1 |
| ILE | 354 | 1 | MET | 697 | 1 |
| VAL | 356 | 1 | LEU | 699 | 1 |
| ILE | 361 | 1 | GLY | 700 | 1 |
| PRO | 365 | 1 | THR | 724 | 1 |
| THR | 377 | 1 | ILE | 726 | 1 |
| ARG | 385 | 1 | LEU | 727 | 1 |
| GLU | 386 | 1 | TYR | 756 | 1 |
| VAL | 387 | 1 | PHE | 759 | 1 |
| | | | LYS | 776 | 1 |
| | | | ALA | 890 | 1 |
| | | | GLY | 891 | 1 |
| | | | LEU | 894 | 1 |

| | | |
|---|---|---|
| ASP | 936 | 1 |
| SER | 937 | 1 |
| SER | 939 | 1 |
| SER | 940 | 1 |
| THR | 941 | 1 |
| ALA | 942 | 1 |
| SER | 943 | 1 |
| GLY | 946 | 1 |
| LYS | 947 | 1 |
| ASP | 950 | 1 |
| ASN | 969 | 1 |
| ALA | 972 | 1 |
| VAL | 991 | 1 |
| ASP | 994 | 1 |
| THR | 998 | 1 |
| LEU | 1001 | 1 |
| GLN | 1005 | 1 |
| GLN | 1010 | 1 |
| LEU | 1012 | 1 |
| ILE | 1013 | 1 |
| ALA | 1016 | 1 |
| ARG | 1019 | 1 |
| SER | 1021 | 1 |
| ALA | 1025 | 1 |
| LYS | 1028 | 1 |
| ARG | 1039 | 1 |
| PHE | 1042 | 1 |
| LYS | 1045 | 1 |
| GLU | 1144 | 1 |
| LEU | 1152 | 1 |
| LYS | 1157 | 1 |
| THR | 1160 | 1 |
| VAL | 1176 | 1 |
| ILE | 1179 | 1 |
| GLN | 1208 | 1 |
| TYR | 1215 | 1 |
| GLY | 1219 | 1 |
| LEU | 1224 | 1 |
| ILE | 1227 | 1 |
| VAL | 1228 | 1 |
| MET | 1237 | 1 |
| CYS | 1241 | 1 |
| SER | 1242 | 1 |
| CYS | 1243 | 1 |
| LEU | 1270 | 1 |

Note: Binding amino acids with the highest occurrence are highlighted in bold.

**Table S22.** Key Binding Amino Acids in the Human CD46 and SARS-CoV-2 Envelope Protein (EP), Membrane Protein (MP), Nucleocapsid Protein (NP), and Spike Protein (SP) Protein-Protein Complexes.

| Amino Acid | Position | Occurrence | Amino Acid | Position | Occurrence |
|---|---|---|---|---|---|
| **CD46↔EP Complex** | | | | | |
| **Human CD46 - 11 amino acids** | | | **SARS-CoV-2 Envelope Protein (EP) - 15 amino acids** | | |
| THR | 188 | 3 | PHE | 20 | 6 |
| TYR | 189 | 3 | ILE | 13 | 5 |
| SER | 190 | 3 | VAL | 5 | 4 |
| CYS | 191 | 3 | THR | 9 | 4 |
| PRO | 193 | 3 | LEU | 12 | 4 |
| PHE | 200 | 3 | SER | 16 | 4 |
| LEU | 202 | 3 | VAL | 17 | 4 |
| GLU | 205 | 3 | LEU | 19 | 4 |
| SER | 206 | 3 | PHE | 23 | 4 |
| THR | 207 | 3 | LEU | 27 | 4 |
| TYR | 247 | 3 | VAL | 29 | 4 |
| | | | LEU | 34 | 4 |
| | | | ARG | 38 | 4 |
| | | | ASN | 45 | 4 |
| | | | ASN | 66 | 4 |
| **CD46↔MP Complex** | | | | | |
| **Human CD46 - 19 amino acids** | | | **MP SARS-CoV-2 – 18 amino acids** | | |
| PHE | 11 | 4 | VAL | 60 | 7 |
| PRO | 12 | 4 | PRO | 59 | 6 |
| TRP | 14 | 4 | ALA | 63 | 6 |
| PHE | 16 | 4 | VAL | 66 | 6 |
| PRO | 17 | 4 | VAL | 70 | 6 |
| LEU | 20 | 4 | ASP | 3 | 5 |
| MET | 24 | 4 | LEU | 56 | 5 |
| VAL | 25 | 4 | LEU | 67 | 5 |
| LEU | 26 | 4 | PHE | 96 | 5 |
| LEU | 27 | 4 | MET | 1 | 4 |
| TYR | 29 | 4 | LEU | 13 | 4 |
| PHE | 31 | 4 | TRP | 20 | 4 |
| GLU | 36 | 4 | LEU | 57 | 4 |
| ASN | 83 | 4 | LEU | 62 | 4 |
| THR | 85 | 4 | TYR | 71 | 4 |
| ILE | 239 | 4 | ILE | 82 | 4 |
| TYR | 247 | 4 | LEU | 90 | 4 |
| THR | 251 | 4 | TRP | 92 | 4 |
| VAL | 269 | 4 | | | |
| **CD46↔NP Complex** | | | | | |
| **Human CD46 – 31 amino acids** | | | **NP SARS-CoV-2 – 31 amino acids** | | |
| ASP | 343 | 5 | PHE | 17 | 4 |
| VAL | 344 | 5 | SER | 21 | 4 |
| TRP | 345 | 5 | SER | 79 | 4 |
| TYR | 247 | 4 | HIS | 145 | 4 |
| SER | 341 | 4 | GLY | 212 | 4 |
| LEU | 342 | 4 | ASP | 216 | 4 |

| | | | | | |
|---|---|---|---|---|---|
| VAL | 346 | 4 | THR | 334 | 4 |
| VAL | 351 | 4 | GLY | 335 | 4 |
| TYR | 132 | 3 | ALA | 336 | 4 |
| ALA | 194 | 3 | ASP | 81 | 3 |
| PRO | 199 | 3 | ASP | 82 | 3 |
| GLU | 205 | 3 | ASN | 140 | 3 |
| TRP | 216 | 3 | THR | 141 | 3 |
| ARG | 218 | 3 | ALA | 211 | 3 |
| PHE | 242 | 3 | ASN | 213 | 3 |
| LYS | 245 | 3 | LEU | 219 | 3 |
| ILE | 338 | 3 | LEU | 223 | 3 |
| LEU | 339 | 3 | ARG | 259 | 3 |
| ASP | 340 | 3 | ARG | 277 | 3 |
| ILE | 347 | 3 | PRO | 279 | 3 |
| ALA | 353 | 3 | GLN | 281 | 3 |
| ILE | 354 | 3 | THR | 282 | 3 |
| VAL | 355 | 3 | GLN | 283 | 3 |
| ALA | 359 | 3 | GLY | 284 | 3 |
| VAL | 360 | 3 | ASN | 285 | 3 |
| TYR | 366 | 3 | PHE | 286 | 3 |
| TYR | 368 | 3 | ILE | 304 | 3 |
| TYR | 378 | 3 | PRO | 309 | 3 |
| THR | 380 | 3 | PHE | 314 | 3 |
| THR | 383 | 3 | TYR | 333 | 3 |
| HIS | 384 | 3 | ILE | 337 | 3 |

**CD46↔SP Complex**

| **Human CD46 - 28 amino acids** | | | **SP SARS-CoV-2 – 16 amino acids** | | |
|---|---|---|---|---|---|
| ARG | 218 | 5 | VAL | 3 | 5 |
| GLN | 122 | 4 | LEU | 5 | 5 |
| GLU | 177 | 4 | MET | 1 | 4 |
| ALA | 219 | 4 | PHE | 2 | 4 |
| LYS | 249 | 4 | PHE | 4 | 3 |
| TYR | 131 | 3 | VAL | 6 | 3 |
| VAL | 178 | 3 | ASP | 198 | 3 |
| PRO | 199 | 3 | GLY | 199 | 3 |
| GLU | 205 | 3 | GLN | 271 | 3 |
| VAL | 215 | 3 | SER | 375 | 3 |
| TRP | 216 | 3 | ASP | 405 | 3 |
| SER | 217 | 3 | ARG | 408 | 3 |
| SER | 240 | 3 | GLY | 502 | 3 |
| PHE | 242 | 3 | VAL | 503 | 3 |
| LYS | 244 | 3 | LEU | 1145 | 3 |
| TYR | 248 | 3 | ASP | 1146 | 3 |
| LYS | 293 | 3 | | | |
| PRO | 295 | 3 | | | |
| LEU | 342 | 3 | | | |
| TRP | 345 | 3 | | | |
| ILE | 347 | 3 | | | |
| VAL | 351 | 3 | | | |
| ALA | 353 | 3 | | | |

| | | |
|---|---|---|
| VAL | 355 | 3 |
| GLY | 357 | 3 |
| ALA | 359 | 3 |
| VAL | 360 | 3 |
| PHE | 389 | 3 |

**Table S23.** Quality Assessment of 3D Models of Human CD46 and SARS-CoV-2 Envelope Protein (EP) Protein-Protein Complexes Obtained Using Different Software Tools.

| Program | Number of Key Binding Amino Acids | | | | Percentage of Key Binding Amino Acids[3] | | | | |
|---|---|---|---|---|---|---|---|---|---|
| | CD46_EP[1] | | EP_CD46[2] | | | | | | |
| | CD46 | EP | CD46 | EP | CD46[4] | EP[5] | CD46_EP[6] | EP_CD46[7] | All[8] |
| ClusPro2 | 11 | 9 | 0 | 0 | 50.0 | 30.0 | 76.9 | 0.0 | 38.5 |
| **GRAMM1** | **11** | **4** | **11** | **4** | **100.0** | **26.7** | **57.7**[9] | **57.7** | **57.7** |
| Hex8 | 0 | 1 | 0 | 2 | 0.0 | 10.0 | 3.8 | 7.7 | 5.8 |
| SwarmDock | 0 | 9 | 0 | 10 | 0.0 | 63.3 | 34.6 | 38.5 | 36.5 |
| ZDock3 | 0 | 12 | 0 | 12 | 0.0 | 80.0 | 46.2 | 46.2 | 46.2 |

Note: 1. In separate subunits of the CD46(receptor)–EP(ligand) complex; 2. In separate subunits of the EP(receptor)–CD46(ligand) complex; 3. Out of the total number of key binding amino acids; 4. In the CD46 subunit of both models; 5. In the EP subunit of both models; 6. In the CD46 (receptor)–EP(ligand) complex; 7. In the EP(receptor)–CD46 (ligand) complex; 8. In both dimeric complexes; 9. Best 3D model.

**Table S24.** Quality Assessment of 3D Models of Human CD46 and SARS-CoV-2 Membrane Protein (MP) Protein-Protein Complexes Obtained Using Different Software Tools.

| Program | Number of Key Binding Amino Acids | | | | Percentage of Key Binding Amino Acids[3] | | | | |
|---|---|---|---|---|---|---|---|---|---|
| | CD46_MP[1] | | MP_CD46[2] | | | | | | |
| | CD46 | MP | CD46 | MP | CD46[4] | MP[5] | CD46_MP[6] | MP_CD46[7] | All[8] |
| ClusPro2 | 15 | 7 | 15 | 8 | 78.9 | 41.7 | 59.5 | 62.2 | 60.8 |
| GRAMM1 | 4 | 5 | 4 | 12 | 21.1 | 47.2 | 24.3 | 43.2 | 33.8 |
| Hex8 | 0 | 0 | 0 | 0 | 0.0 | 0.0 | 0.0 | 0.0 | 0.0 |
| SwarmDock | 4 | 14 | 4 | 13 | 21.1 | 75.0 | 48.6 | 45.9 | 47.3 |
| **ZDock3** | **15** | **14** | **15** | **14** | **78.9** | **77.8** | **78.4**[9] | **78.4** | **78.4** |

Note: 1. In separate subunits of the CD46(receptor)–MP(ligand) complex; 2. In separate subunits of the MP(receptor)–CD46(ligand) complex; 3. Out of the total number of key binding amino acids; 4. In the CD46 subunit of both models; 5. In the MP subunit of both models; 6. In the CD46 (receptor)–MP(ligand) complex; 7. In the MP(receptor)–CD46 (ligand) complex; 8. In both dimeric complexes; 9. Best 3D model.

**Table S25.** Quality Assessment of 3D Models of Human CD46 and SARS-CoV-2 Nucleocapsid Protein (NP) Protein-Protein Complexes Obtained Using Different Software Tools.

| Program | Number of Key Binding Amino Acids | | | | Percentage of Key Binding Amino Acids[3] | | | | |
|---|---|---|---|---|---|---|---|---|---|
| | CD46_NP[1] | | NP_CD46[2] | | | | | | |
| | CD46 | NP | CD46 | NP | CD46[4] | NP[5] | CD46_NP[6] | NP_CD46[7] | All[8] |
| ClusPro2 | 0 | 17 | 0 | 17 | 0.0 | 54.8 | 27.4 | 27.4 | 27.4 |
| GRAMM1 | 12 | 6 | 18 | 12 | 48.4 | 29.0 | 29.0 | 48.4 | 38.7 |
| Hex8 | 4 | 2 | 4 | 6 | 12.9 | 12.9 | 9.7 | 16.1 | 12.9 |
| SwarmDock | 21 | 22 | 5 | 4 | 41.9 | 41.9 | 69.4 | 14.5 | 41.9 |
| **ZDock3** | **20** | **8** | **20** | **8** | **64.5** | **25.8** | **45.2**[9] | **45.2** | **45.2** |

Note: 1. In separate subunits of the CD46(receptor)–NP(ligand) complex; 2. In separate subunits of the NP(receptor)–CD46(ligand) complex; 3. Out of the total number of key binding amino acids; 4. In the CD46 subunit of both models; 5. In the NP subunit of both models; 6. In the CD46 (receptor)–NP(ligand) complex; 7. In the NP(receptor)–CD46 (ligand) complex; 8. In both dimeric complexes; 9. Best 3D model.

**Table S26.** Quality Assessment of 3D Models of Human CD46 and SARS-CoV-2 Spike Protein (SP) Protein-Protein Complexes Obtained Using Different Software Tools.

| Program | Number of Key Binding Amino Acids | | | | Percentage of Key Binding Amino Acids3 | | | | |
|---|---|---|---|---|---|---|---|---|---|
| | CD46_SP[1] | | SP_CD46[2] | | | | | | |
| | CD46 | SP | CD46 | SP | CD46[4] | SP[5] | CD46_SP[6] | SP_CD46[7] | All[8] |
| ClusPro2 | 12 | 2 | 11 | 2 | 41.1 | 12.5 | 31.8 | 29.5 | 30.7 |
| GRAMM1 | 8 | 7 | 2 | 3 | 17.9 | 31.3 | 34.1 | 11.4 | 22.7 |
| Hex8 | 9 | 0 | 6 | 0 | 26.8 | 0.0 | 20.5 | 13.6 | 17.0 |
| SwarmDock | 14 | 9 | 8 | 5 | 39.3 | 43.8 | 52.3 | 29.5 | 40.9 |
| **ZDock3** | **10** | **13** | **10** | **13** | **35.7** | **81.3** | **52.3**[9] | **52.3** | **52.3** |

Note: 1. In separate subunits of the CD46(receptor)–SP(ligand) complex; 2. In separate subunits of the SP(receptor)–CD46(ligand) complex; 3. Out of the total number of key binding amino acids; 4. In the CD46 subunit of both models; 5. In the SP subunit of both models; 6. In the CD46 (receptor)–SP(ligand) complex; 7. In the SP(receptor)–CD46 (ligand) complex; 8. In both dimeric complexes; 9. Best 3D model.

**Table S27.** Key Binding Amino Acids in the Interaction Regions of Subunits in the Best Valid 3D Models of Human CD46 and SARS-CoV-2 Envelope Protein (EP), Membrane Protein (MP), Nucleocapsid Protein (NP), and Spike Protein (SP) Protein-Protein Complexes.

| Model | Key Binding Amino Acids | |
|---|---|---|
| | CD | Second Subunit |
| CD46_EP_GRAMM1 | THR188, TYR189, SER190, CYS191, PRO193, PHE200, LEU202, GLU205, SER206, THR207, TYR247 | ILE13, VAL17, PHE20, VAL29 |
| CD46_MP_ZDock3 | PHE11, PRO12, TRP14, PHE16, PRO17, LEU20, MET24, VAL25, LEU26, LEU27, TYR29, PHE31, GLU36, ASN83, THR85 | MET1, ASP3, LEU56, LEU57, PRO59, VAL60, LEU62, ALA63, VAL66, LEU67, VAL70, TYR71, TRP92, PHE96 |
| CD46_NP_ZDock3 | ILE338, SER341, LEU342, ASP343, VAL344, TRP345, VAL346, ILE347, VAL351, ALA353, ILE354, VAL355, ALA359, VAL360, TYR366, TYR368, TYR378, THR380, THR383, HIS384 | PHE17, SER21, ALA211, GLY212, ASN213, ASP216, LEU219, LEU223 |

| | | |
|---|---|---|
| CD46_SP_ZDock3 | GLU177, VAL178, PRO199, GLU205, ARG218, SER240, TYR248, LYS249, LYS293, PRO295 | MET1, PHE2, VAL3, PHE4, LEU5, VAL6, ASP198, GLY199, SER375, ASP405, ARG408, GLY502, VAL503 |
